# Biapenem Inactivation by B2 Metallo β-Lactamases: Energy Landscape of the Post-Hydrolysis Reactions

**DOI:** 10.1371/journal.pone.0030079

**Published:** 2012-01-12

**Authors:** Domenico L. Gatti

**Affiliations:** 1 Department of Biochemistry and Molecular Biology, Wayne State University School of Medicine, Detroit, Michigan, United States of America; 2 Cardiovascular Research Institute, Wayne State University School of Medicine, Detroit, Michigan, United States of America; The Scripps Research Institute, United States of America

## Abstract

**Background:**

The first line of defense by bacteria against *β*-lactam antibiotics is the expression of β-lactamases, which cleave the amide bond of the β-lactam ring. In the reaction of biapenem inactivation by B2 metallo β-lactamases (MβLs), after the β-lactam ring is opened, the carboxyl group generated by the hydrolytic process and the hydroxyethyl group (common to all carbapenems) rotate around the C5–C6 bond, assuming a new position that allows a proton transfer from the hydroxyethyl group to C2, and a nucleophilic attack on C3 by the oxygen atom of the same side-chain. This process leads to the formation of a bicyclic compound, as originally observed in the X-ray structure of the metallo β-lactamase CphA in complex with product.

**Methodology/Principal Findings:**

QM/MM and metadynamics simulations of the post-hydrolysis steps in solution and in the enzyme reveal that while the rotation of the hydroxyethyl group can occur in solution or in the enzyme active site, formation of the bicyclic compound occurs primarily in solution, after which the final product binds back to the enzyme. The calculations also suggest that the rotation and cyclization steps can occur at a rate comparable to that observed experimentally for the enzymatic inactivation of biapenem only if the hydrolysis reaction leaves the N4 nitrogen of the β-lactam ring unprotonated.

**Conclusions/Significance:**

The calculations support the existence of a common mechanism (in which ionized N4 is the leaving group) for carbapenems hydrolysis in all MβLs, and suggest a possible revision of mechanisms for B2 MβLs in which the cleavage of the β-lactam ring is associated with or immediately followed by protonation of N4. The study also indicates that the bicyclic derivative of biapenem has significant affinity for B2 MβLs, and that it may be possible to obtain clinically effective inhibitors of these enzymes by modification of this lead compound.

## Introduction

The first line of defense by bacteria against *β*-lactam antibiotics is the expression of β-lactamases, which cleave the amide bond of the β-lactam ring and inactivate the antibiotics [1]. These enzymes are grouped in four classes (A to D) according to sequence homology and mechanism [2], [3], [4], [5]. In class A, C, and D an active site serine is acylated by the antibiotic followed by deacylation and opening of the β-lactam ring. Class B enzymes are metallo-proteins requiring one or two Zn^2+^ ions for activity [5]. Metallo β-lactamases (MβLs) can degrade a broader spectrum of β-lactams than the serine-types, and are particularly efficient as carbapenemases. This last characteristic is alarming because carbapenems are the antibiotics with the largest spectrum of activity, and are stable to hydrolysis by most of the serine β-lactamases [6]. The danger associated with the spread of MβLs is compounded by the absence of clinically relevant inhibitors [6], [7].

Based on their sequence heterogeneity MβLs are grouped into three subclasses (B1 to B3) [5]. Although the sequence identity is low, comparison of the tertiary structure of enzymes from the three subclasses reveals a common αβ/βα sandwich fold, and structure based sequence alignment has produced a standard numbering that allows easy comparison of the various sequences [8]. All MβLs possess two potential Zn^2+^ sites, but the three subclasses display different Zn^2+^ occupancies and coordination environments [5]. In B1 enzymes one Zn is coordinated by His116, His118, His196 (Site 1 or Zn1), and the other Zn by Asp120, Cys221, His263 (Site 2 or Zn2). A water molecule (or hydroxide ion) bridges the two Zn^2+^ ions. In B2 enzymes Site 1 is incomplete (having only His118 and His196) and without metal, and only Site 2 is occupied. In B3 enzymes Site 1 is unchanged, while at Site 2 His121 replaces Cys221 as a Zn ligand. B1 and B3 enzymes have a broad substrate profile and maximum activity as di-zinc species [9]. In contrast, B2 enzymes have a relatively narrow substrate profile (almost exclusively carbapenems) [9], and exhibit maximal activity when bound to only one Zn, the binding of a second Zn ion being inhibitory [10], [11].

**Figure 1:**
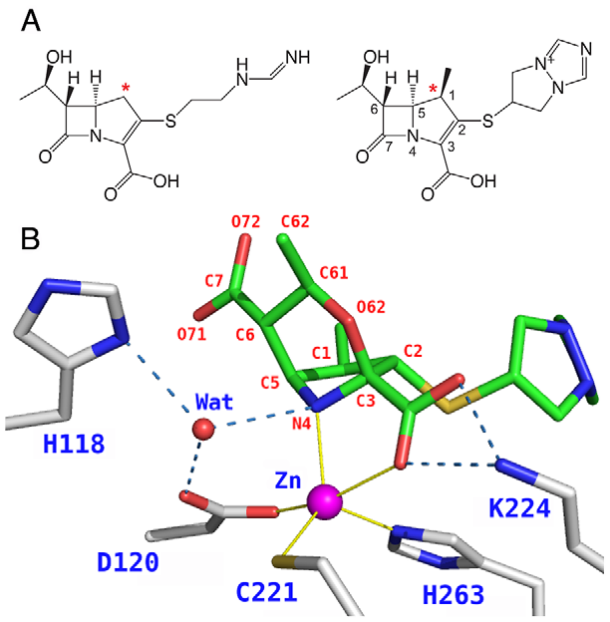
CphA sustrates and products. **A.** Two carbapenems: imipenem (left), biapenem (right). An asterisk marks the carbon atom that replaces the sulfur of penicillins. Biapenem differs from imipenem for the presence of a C-1β methyl group and a C-2 σ-symmetric (6,7-dihydro-5*H*-pyrazolo[1,2-*a*][1], [2], [4] triazolium-6-yl)thio group. Notice the C6 hydroxyethyl group common to all carbapenems. **B.** Bicyclic derivative of biapenem bound in the active site of CphA (PDB entry 1X8I). His118, Asp120 and biapenem N4 are shown here as unprotonated, and thus the hydrogen bond pattern of Wat is undefined; the actual ionization state of these groups inside the bacterial cell may be different. Zn^2+^ coordination and hydrogen bonds are shown as thin yellow lines and dashed blue lines.

**Figure 2:**
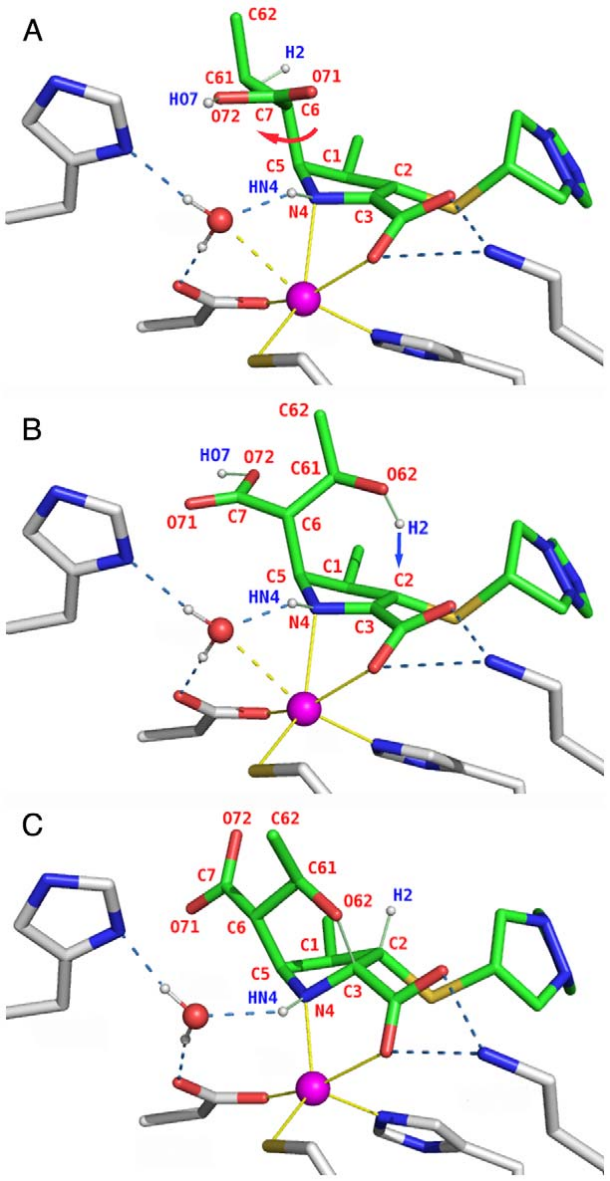
Formation of the bicyclic derivative of biapenem. **A.** QM/MM optimized model of the active site of CphA in complex with hydrolyzed biapenem (“Conformation A” in the metadynamics analysis of the hydroxyethyl group rotations described later on). In the configuration shown here both N4 and the C6 carboxylate are protonated (atoms HN4 and HO7). A water molecule (originating from bulk solvent after the hydrolysis reaction is completed) is hydrogen bonded to Asp120 and loosely coordinated to Zn^2+^ (dashed yellow bond). Zn^2+^ has only five strong ligands, in agreement with spectroscopic data [29]. A red arrow indicates the rotation of the C6 carboxylate and hydroxyethyl moieties (the latter only partially visible) required to generate the open-ring form shown in the next panel. **B.** Conformation of hydrolyzed biapenem that precedes the formation of the bicyclic compound (“Conformation B” in the metadynamics analysis of the hydroxyethyl group rotations). A blue arrow indicates the proton transfer from O62 to C2 required to generate the bicyclic compound. **C.** Active site of CphA in complex with the bicyclic derivative of biapenem. The C2–H2 and O62–C3 bonds formed during the rearrangement are shown as thin green lines. N4 is protonated.

Based on structural, biochemical and computational studies a general mechanism has been proposed for MβLs, in which the positive charge of one or both Zn ions and the presence of likely proton acceptors (His or Asp) favor the deprotonation of the nearby water molecule, generating the hydroxide ion that attacks the carbonyl carbon of the β-lactam ring [12], [13], [14], [15], [16], [17], [18], [19], [20], [21], [22], [23], [24], [25], [26], [27]. However, each enzyme has slightly different mechanistic features. In this study we focus on the post-hydrolysis steps of biapenem (**Fig. 1A**) inactivation by CphA from *Aeromonas hydrophila*, a B2 type MβL. CphA is a strict carbapenemase with negligible activity against penicillins and cephalosporins. The structure of N220G CphA, which retains ∼½ the activity of the wild type enzyme, was determined by Garau *et al.* [19] in complex with a form of hydrolyzed biapenem that has undergone a molecular rearrangement such that the oxygen atom (O62) of the C6 hydroxyethyl moiety forms a 6-membered ring (N4-C5-C6-C61-O62-C3) that replaces the original β-lactam ring (**Fig. 1B**). In that study the authors interpreted the electron density of the ligand as representative of an unprotonated intermediate of the reaction rather than the product. According to their interpretation, cleavage of the β-lactam ring would initially leave the N4 nitrogen of biapenem ionized (negatively charged). Then, after the bicyclic compound is formed, donation of a proton to the N4 nitrogen by the nearby water would lead to the opening of the 6-membered ring with formation of the final hydrolyzed and protonated product. Most recently, Wu *et al.* [28] examined the energetics of the cyclization reaction by quantum-mechanical/molecular-mechanical (QM/MM) calculations and confirmed the mechanism proposed by Garau *et al.* [19], but concluded that the bicyclic compound is only an intermediate or product in a minor pathway, as the main pathway would lead instead to direct protonation of the N4 nitrogen of the hydrolyzed form of biapenem. However, this conclusion does not easily explain the observation that only the bicyclic compound was observed bound in the active site of CphA, or the fact that a similar bicyclic compound was identified as the main product also in the hydrolysis of imipenem/meropenem by the B2 MβL ImiS from *Aeromonas* V*eronii b*V. *Sobria* [29]. To explain their result with the ImiS enzyme the authors proposed a different mechanism for the cyclization reaction as shown in **Fig. 2** (applied here to biapenem rather than imipenem): after a water molecule attacks the carbonyl carbon (C7) of the β-lactam ring, the ring is opened and the leaving nitrogen N4 is protonated (**Fig. 2A**); the active site is repopulated by water originating from the bulk solvent. Then, the carboxyl group generated by the hydrolytic process and the hydroxyethyl group rotate around the C5–C6 bond, assuming the position observed in the experimental complex (**Fig. 2B**). Final proton transfer from the hydroxyethyl group to C2, and nucleophilic attack on C3 by the oxygen atom (O62) of the same side-chain generate the bicyclic product (**Fig. 2C**).

The two mechanisms proposed respectively for CphA and ImiS essentially differ by the fact that in one case the reaction (along the minor pathway) starts with an open ring form of biapenem with N4 deprotonated, goes through a cyclic intermediate, and ends with hydrolyzed biapenem with N4 protonated, while in the other case the reaction (along the main pathway) starts with hydrolyzed imipenem with N4 protonated and ends with the protonated cyclic product.

The important issue here is that if the bicyclic compound is the main final product of carbapenems hydrolysis by B2 MβLs, then the compound itself or the transition state(s) that lead to it may represent valuable starting points for the design of effective inhibitors of this class of MβLs. Furthermore, since the reactant state (RS) of the cyclization reaction is the product state (PS) of the hydrolysis reaction, discrimination between the two mechanisms has relevance not only for the formation of the bicyclic compound, but also for the correct mechanism of the hydrolysis reaction. For this reason, in this study we investigated both mechanisms using a combination of metadynamics simulations and QM/MM simulations based on density functional theory (DFT).

## Results

### Post-hydrolysis reactions in water

The uncatalyzed formation of the bicyclic compound from hydrolyzed biapenem was simulated inside a sphere of water molecules of 26 Å radius reproducing the actual concentration of water at 1 atm and 298.15 K. Hydrolyzed biapenem was in the conformation that ensues upon rotation of the hydroxyethyl group around the C5–C6 bond (like in **Fig. 2B**), but two separate QM/MM simulations were carried out with N4 protonated or deprotonated as these are the two possible chemical forms of the RS for the post-hydrolysis reaction. Potential energy surfaces (PESs) were constructed using QM/MM relaxed scans (see Methods) on a two-dimensional grid employing the forming C–H bond between the hydroxyl hydrogen and C2 of biapenem and the forming C–O bond between the hydroxyethyl oxygen and C3 of biapenem as reaction coordinates. Between 200 and 400 points were obtained for each PES, and at each point a full geometry optimization was carried out, allowing for the reorganization of the solvent along the reaction coordinate. Thermochemical properties at the TSs and stationary points on the PESs were calculated from the vibrational properties of the states as described in the Methods section.

**Figure 3:**
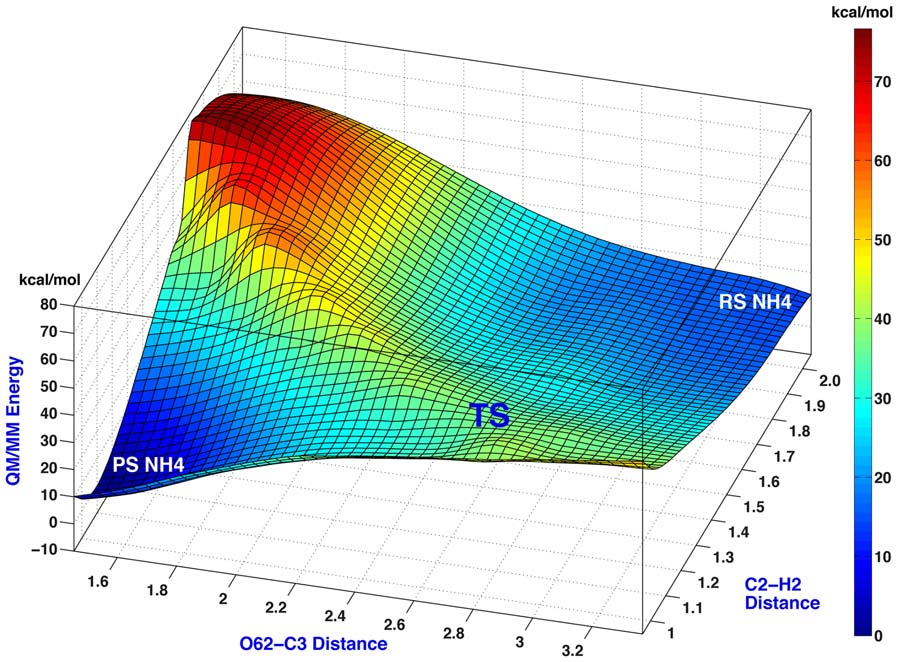
PES of the non-enzymatic formation of the bicyclic derivative of biapenem when N4 is protonated. The PES is defined by two reaction coordinates: the forming C2–H2 bond between the hydroxyl hydrogen and C2 and the forming C3–O62 bond between the hydroxyl oxygen and C3. The position of the only TS is marked on the surface. QM/MM energies are in kcal/mol. Colors on the PESs reflect the QM/MM energy levels, as represented in the reference bar on the side.

The PES of the bicyclic compound formation with N4 protonated is shown in **Fig. 3**. A single TS is visible on the PES and was confirmed by vibrational analysis. At the TS the C2–H bond length is ∼1.2 Å and the distance between the hydroxyethyl oxygen and C3 is ∼2.9 Å. Thus, the rate-limiting step of the reaction is the proton transfer from the hydroxyl oxygen (O62 in **Fig. 2B**) to C2. Past the TS, subsequent formation of the C3–O62 bond is downhill.

**Figure 4:**
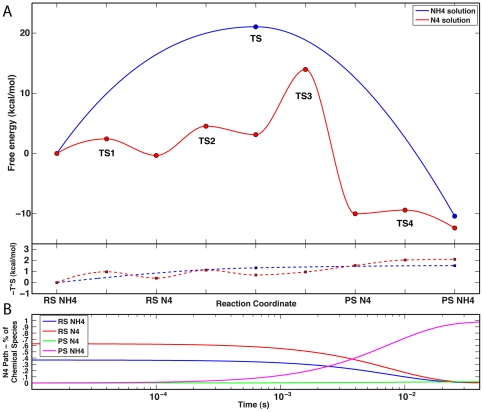
Free energy profiles of the cyclization reaction in solution. **A.** The profile of the reaction with N4 protonated (corresponding to the PES of **Fig. 3**) is shown in the upper quadrant as a blue line. The reaction coordinate axis is in arbitrary units and the reactant and product states are marked as RS NH4 and PS NH4, respectively. The profile of the reaction with N4 deprotonated is shown as a red line in the upper quadrant. The actual reactant and product states of the cyclization reaction (corresponding to PES II in **Fig. 5**) are marked as RS N4 and PS N4, respectively. In order to place this reaction on the same energy scale as the reaction with N4 protonated, two additional steps were calculated representing the protonation of RS N4 to RS NH4 (PES I in **Fig. 5**), and the protonation of PS N4 to PS NH4 (PES III in **Fig. 5**). Circles on the profiles mark the positions along the reaction coordinate (TSs and stationary points) where thermochemical properties were calculated with a vibrational analysis. All other values reflect only a shape-preserving interpolation between the calculated points. Changes in the entropic contribution (−T*S) to the free energy curves are shown in the lower quadrant with red and blue dashed lines and square markers for the NH4 and N4 pathways, respectively. **B.** Time course of the reaction corresponding to the red trace in panel A, starting from 100% hydrolyzed biapenem with N4 ionized (RS N4 in panel A). The almost instantaneous (between 0 and 10^−5^ s) protonation of RS N4 to RS NH4 (due to the very low barrier at TS1) is not shown.

The free energy profile of this reaction is shown as a blue line in the upper quadrant of **Fig. 4A**. Changes in the entropic contributions (−T*S) to the free-energy curve are shown in the lower quadrant of the figure. The reaction is spontaneous under standard conditions (ΔG^0^ = −10.41 kcal/mol), with a barrier (ΔG^‡^ = 21.05 kcal/mol) corresponding to a forward rate constant *k*≅*k*′≅0.002 s^−1^. Although only one TS is present, the reaction is not strictly concerted in a chemical sense as the TS is much closer to the formation of the C2–H bond than to the formation of the C3–O bond.

**Figure 5:**
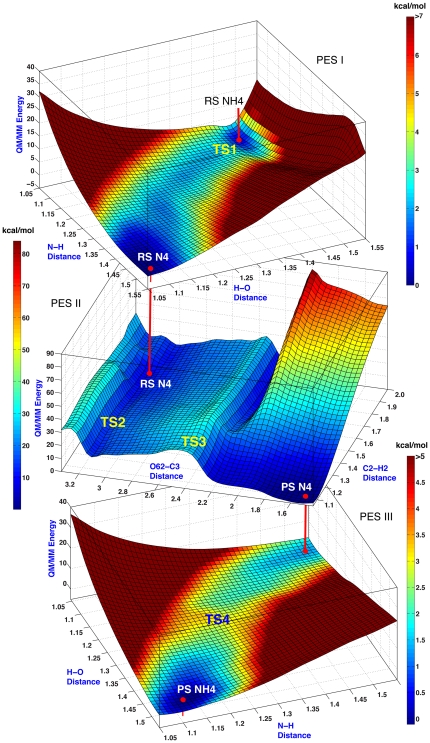
PESs of the non-enzymatic formation of the bicyclic derivative of biapenem when N4 is deprotonated. The reaction path corresponding to the red trace in **Fig. 4** spans three separate PESs: red vertical lines connect the equivalent phase-space points of each PES (the jump points from one PES to another). PES I corresponds to a proton transfer (TS1) from N4 of hydrolyzed biapenem to a hydroxide ion. PES II corresponds to the proton transfer from the hydroxyl oxygen to C2 (TS2) and the formation of the C3–O bond (closure of the 6-membered ring, TS3). PES III corresponds to the reprotonation of N4 from water (TS4). QM/MM energies are in kcal/mol. Colors on the PESs reflect the QM/MM energy levels, as represented in the reference bars on the side of each PES.

The PES of the bicyclic compound formation with N4 deprotonated is shown in **Fig. 5** (PES II). To place this PES on the same free energy scale as the PES with N4 protonated (shown in **Fig. 3**) two additional PESs were calculated, representing respectively the protonation of N4 of hydrolyzed biapenem (**Fig. 5**, PES I), and the protonation of N4 of the bicyclic compound (**Fig. 5**, PES III). In both cases a water molecule was the proton donor. Notably, protonation of N4 of the bicyclic compound did not lead in this simulation to the opening of the oxazine ring as predicted by Garau *et al.* [19]. The PES of the cyclization reaction alone (**Fig. 5**, PES II) is more complex than that of the same reaction with N4 protonated (**Fig. 3**): two distinct TSs were identified by vibrational analysis, one corresponding to the proton transfer from the hydroxyl oxygen to C2 (TS2) and the other corresponding to the formation of the C3–O bond (closure of the 6-membered ring, TS3). Thus, if N4 is deprotonated, the cyclization reaction occurs stepwise, and the rate-limiting step is the formation of the C3–O bond (TS3).

The free energy profile of the entire path (including the two protonation steps corresponding to PES I and III) is shown as a red line in **Fig. 4A**. Whether the reaction starts with protonated N4 (RS NH4 in **Fig. 4A**) or unprotonated N4 (RS N4 in **Fig. 4A**), it is expected to occur spontaneously under standard conditions (ΔG^0^ = −12.36 kcal/mol). The highest barrier in the reaction (ΔG^TS3-RSN4^ = 14.3 kcal/mol) corresponds to a maximal rate constant of 210 s^−1^; however, the net forward rate constant calculated by solving the ODEs that describe the entire path is a little less (*k*′ = ∼128 s^−1^). Simulation of the entire reaction time-course reveals that, if the reaction starts with 100% hydrolyzed biapenem deprotonated at N4 (RS N4), a transient equilibrium is reached almost instantly (*k* = 6×10^10^ s^−1^), in which 37% of hydrolyzed biapenem is protonated at N4 (RS NH4 in **Fig. 4B**); the final equilibrium with zero net fluxes, in which 98% of all biapenem is in bicyclic form with N4 protonated and 2% in bicyclic form with N4 deprotonated (**Fig. 4B**), is reached within 30 ms. These results are consistent with the well-known fact that the anionic (at N4) open ring form of β-lactams has a very high p*K*_a_ and is mostly protonated in solution in the absence of metal ions [12], [14], [30], [31]. What is shown here however is that hydrolyzed biapenem with the hydroxyethyl group rotated (as in **Fig. 2B**) is expected to be completely converted to its bicyclic form with N4 protonated (RS NH4 in **Fig. 4A**) in less then 30 msec, following a path in which N4 is transiently ionized.

In principle, the ΔG^0^ value (−12.36 kcal/mol) calculated for the path with N4 deprotonated (red line in **Fig. 4A**) should be identical to the ΔG^0^ value (−10.41 kcal/mol) calculated for the path with N4 protonated (blue line in **Fig. 4A**), because the end points represent the same chemical entities in solution. The similarity (within an acceptable error of 1–2 kcal/mol) of the calculated reaction energies for the two pathways (the blue and red lines in **Fig. 4A**, respectively), despite the fact that the two simulation ensembles were created and equilibrated independently, attests to the accuracy of the calculation. Of particular interest is the fact that the overall reaction barrier is much smaller for the unprotonated pathway, and of the same magnitude as the barrier measured experimentally (∼14 kcal/mol) for the reaction of biapenem inactivation catalyzed by CphA [19]. Thus, the existence of a low energy path (red line in **Fig. 4A**) that starts from hydrolyzed biapenem with the hydroxyethyl group rotated (as shown in **Fig. 2B**) and progresses through intermediates with N4 deprotonated, will pull the equilibrium toward the formation of the bicyclic compound (with N4 protonated) at approximately the same rate as the enzymatic hydrolysis of the β-lactam ring.

### Post-hydrolysis reactions in the enzyme

While the simulations presented in the previous section suggest that formation of the bicyclic derivative of biapenem (and possibly other carbapenems) could form at a significant rate in solution without enzyme intervention, there is also experimental evidence that a similar compound is the final product of the enzymatic reaction catalyzed by B2 MβL ImiS from *Aeromonas* V*eronii b*V. *Sobria*; furthermore, this bicyclic derivative was shown to be kinetically competent as a product inhibitor of ImiS with a *K*_i_ around 50 µM [29]. Therefore, it is of interest to determine if the formation of the bicyclic derivative of biapenem (or other carbapenems) could be accelerated by B2 MβLs with respect to the same reaction in solution. For this purpose we have simulated the cyclization reaction using as scaffold the X-ray structure of CphA from *Aeromonas hydrophila* (PDB entry 1X8I), which was determined with the bicyclic compound bound in the active site [19]. First, the reaction was reversed manually, by breaking the C3–O bond and transferring the C2 hydrogen to the hydroxyethyl oxygen. This operation generated the form of hydrolyzed biapenem that ensues upon rotation of the hydroxyethyl group around the C5–C6 bond (like in **Fig. 2B**). The ionization and/or orientation of key chemical groups in the active site were also modified in order to produce a set of different configurations of the protein in the reactant state (RS). Finally each RS was optimized by QM/MM, and the reaction was driven toward the formation of the bicyclic compound by linear relaxed scans of the C2–H and C3–O bond lengths. At each point along the scans a full geometry optimization was carried out to allow the protein and the solvent to reorganize in response to the changes of charge and topology taking place in the bound antibiotic. In order to compare directly the reactions simulated in the enzyme with those calculated in solution, in all cases the reaction product was the bicyclic derivative of biapenem with N4 protonated (although intermediate steps along the path might have N4 deprotonated).

Eight different configurations of the active site were initially considered (**Table 1**), varying in the protonation state of biapenem N4 (N4^−^, NH4), in the orientation, tautomerization and protonation state of His196 (HID, neutral His with proton on the δ nitrogen; HIE, neutral His with proton on the ε nitrogen; rHIE, HIE rotated by 180° around the Cβ-Cγ bond; HIP, positively charged His), the protonation state of Asp120 (O^−^, OH), the protonation state (H_1_O^−^, H_1_OH_2_) and hydrogen bonds (H-bond acceptors from water H_1_ and H_2_, and H-bond donors to water O) of the water molecule situated near the Zn ion. Four configurations (numbered 5 through 8 in **Table 1**) were discarded based on low-resolution (0.25 Å steps) scans of the C2–H coordinate which revealed large positive values of either the reaction energy (>10 kcal/mol) or the reaction barrier (>25 kcal/mol). The other four configurations (numbered 1 through 4 in **Table 1**) were scored as “promising” based on the initial scan, and were analyzed in further detail. Refinement of the energy profile of these conditions was carried out by driving the reaction in smaller steps (0.05–0.1 Å), with full geometry optimization at each step, first along the C2–H coordinate and then along the C3–O coordinate. A vibrational analysis was carried out for each stationary point (SP) and TS, and the association of each TS with the two neighbor SPs was verified by an Intrinsic Reaction Coordinate (IRC) analysis in the forward and reverse direction using the method of Gonzales and Schlegel [32], [33], [34].

**Table 1:** Configurations of the active site of CphA studied by QM/MM.

Enzyme Configuration	N4^BIA^	H196	D120	WATER	H-bond acceptor from WATER H_1_	H-bond acceptor from WATER H_2_	H-bond donor to WATER O	Marker color in Fig. 6A
1	NH4	HIE	O^−^	H_1_OH_2_	D120(O)	H118(ND1)	BIA(HN4)	GREEN
2	NH4	HIE	O^−^	H_1_O^−^	D120(O)		BIA(HN4)	MAGENTA
3	N4^−^	HIE	OH	H_1_OH_2_	H118(ND1)	BIA(N4)	D120(HD2)	YELLOW
4	N4^−^	rHIP	OH	H_1_OH_2_	H118(ND1)	BIA(N4)	D120(HD2) H196(HE2)	CYAN
5	N4^−^	HIE	O^−^	H_1_OH_2_	D120(O)	H118(ND1)		
6	N4^−^	rHIE	O^−^	H_1_OH_2_	D120(O)	H118(ND1)	H196(HE2)	
7	N4^−^	rHIE	O^−^	H_1_OH_2_	D120(O)	H118(ND1)	H196(HE2)	
8	NH4	HID	OH	H_1_OH_2_	H118(ND1)	H196(NE2)	D120(HD2) BIA(HN4)	

**Figure 6:**
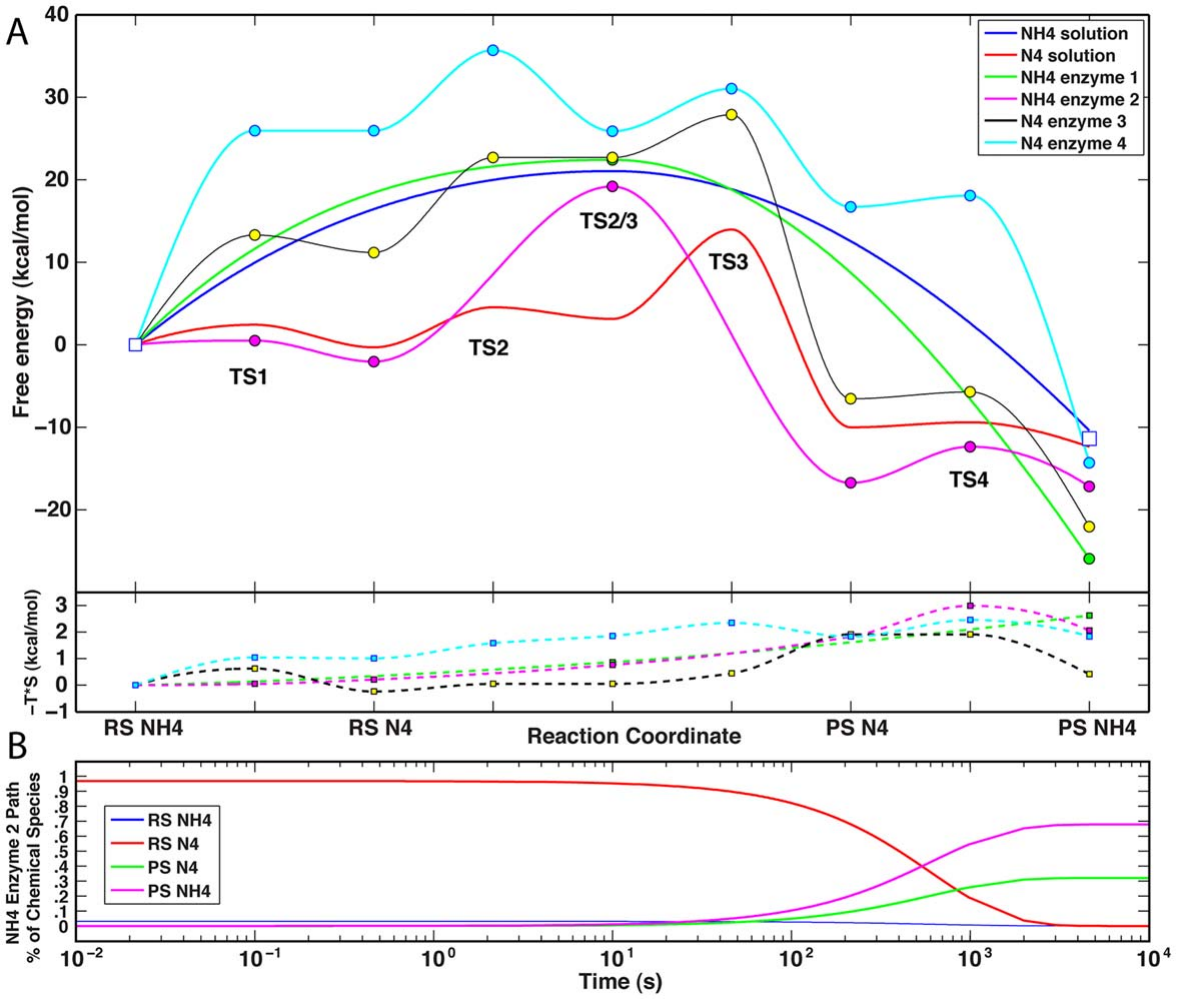
Free energy profiles of the cyclization reaction in the enzyme. **A.** The free energy profiles for the formation of the bicyclic compound in four configurations of the enzyme∶hydrolyzed biapenem complex (continuous lines and colored circles) are superimposed on the profiles of the same reaction in water with protonated and unprotonated N4 (blue and red lines, respectively), as already shown in **Fig. 4A**. All the profiles have their origin coincident with that of protonated reactant in solution (clear square on the left side). The difference in the free energy of the protonated product (PS NH4) between the catalyzed (cyan, magenta, yellow, and green circles) and the uncatalyzed reactions (clear square on the right side) reflects the difference in the free energy of binding (ΔG^bind^) of the reactant versus the product. Changes in the entropic contribution (−T*S) to the free energy curves are shown in the lower quadrant with dashed lines and square markers of the corresponding color. **B.** Time course of the reaction corresponding to the magenta trace in panel A (enzyme configuration No. 2 in **Table 1**), starting from 100% hydrolyzed biapenem with N4 ionized (RS N4 in panel A).

**Figure 7:**
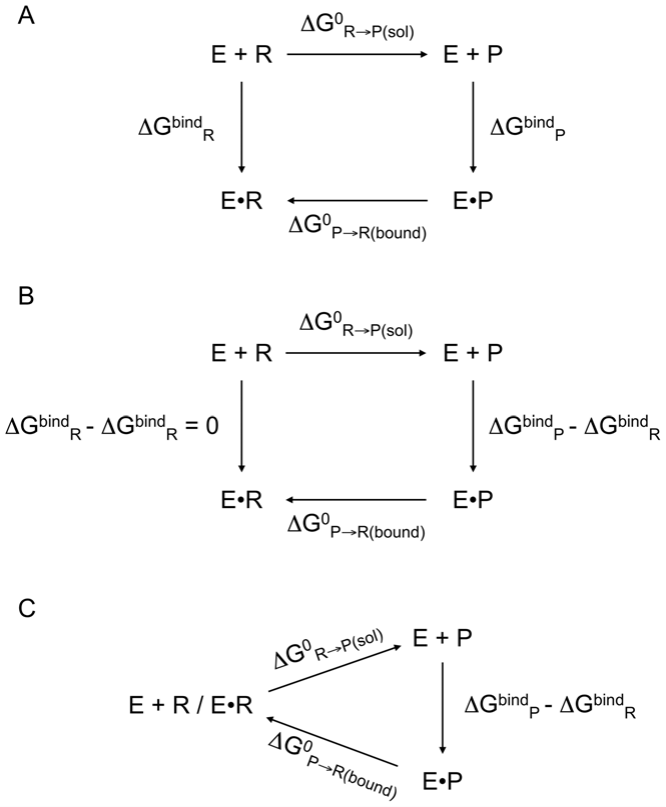
Thermodynamic cycles for the cyclization reactions of hydrolyzed biapenem. **A.** Thermodynamic cycle relating the energy of the cyclization reaction in solution with the energy of the same reaction in the enzyme active site. E•R and E•P are the enzyme∶reactant and the enzyme∶product complexes, respectively. **B.** Intermediate step leading to the collapsed thermodynamic cycle shown in panel C. **C.** Collapsed thermodynamic cycle in which the same energy quantity (ΔG^bind^_R_) has been subtracted from the vertical legs of the cycle shown in panel A. The difference between the catalyzed (lower branch) and the uncatalyzed reaction (upper branch) reflects the difference in the free energy of binding (ΔG^bind^) of the product versus the reactant (vertical leg). While this cycle does not represent a real physical entity, it provides a rationalization for the convention adopted in **Figure 6**, according to which all the free energy profiles of the reaction in the enzyme were placed with their origin coincident with that of the reaction in solution.

The complete free energy profile (with G values derived from vibrational analyses) for the formation of the bicyclic compound in the selected four configurations of the enzyme is shown in the upper quadrant of **Fig. 6A** (continuous lines and colored circles) superimposed on the profiles of the same reaction in water with protonated and unprotonated N4 (blue and red lines without markers, respectively) as already shown in **Fig. 4A**. The entropic contributions (−T*S) to the free-energy curves are shown in the lower quadrant of **Fig. 6A**. Two enzyme simulations (No. 1 and 2 in **Table 1**) started from hydrolyzed biapenem with N4 protonated (RS NH4) and two (No. 3 and 4 in **Table 1**) from biapenem with N4 deprotonated (RS N4). However, for configurations No. 3 and 4 we also calculated the part of the energy profile that represents a direct protonation of N4 (RS N4 ⇒ RS NH4) without going through a cyclic intermediate; therefore, all the profiles start with RS NH4 and end with PS NH4. There are two possible entry points to each profile from the previous step of hydrolysis of the β-lactam ring, one representing the conclusion of the hydrolysis reaction with protonated N4 (entry point RS NH4), and the other representing the conclusion of the hydrolysis reaction with deprotonated N4 (entry point RS N4). The exact position of the enzyme curves with respect to the solution curves is not known because the absolute values of the binding energy of the reactant(s) (protonated or deprotonated hydrolyzed biapenem) and the product (protonated bicyclic derivative) for the specific forms of the enzyme under consideration are not known. Our convention is to set the RS NH4 state of all the enzyme profiles coincident with the RS NH4 state in the solution profiles; the rationale for this convention is shown in **Fig. 7**. In **Fig. 7A** we have drawn a thermodynamic cycle relating the reaction in solution to the reaction in the enzyme via two legs representing the binding energies of the reactant and product to the enzyme. From this cycle we see that:
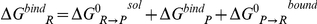
rearranging:
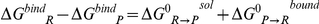
and finally:
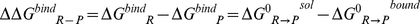
(1)This algebraic relationship can be derived also by subtracting the quantity ΔG^bind^_R_ from both vertical legs of the cycle as shown in **Fig. 7B**: the subtraction is allowed because the two legs belong to different paths of the cycle that share the same start and end points. As a consequence the left leg of the cycle collapses and the energy levels of the reactant in solution and in the enzyme become coincident. The collapsed cycle shown in **Fig. 7C** corresponds to the convention adopted in **Fig. 6A**. From this cycle we can easily verify that:
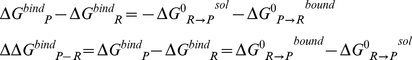
(2)which is completely equivalent to (1). Alternative collapsed cycles can be constructed (see **Figs. S1,S2,S3**) leading to different equally valid conventions (for example, we could place the right end side rather than the left end side of the energy profiles at the same level), which however all correspond to the same algebraic relationship between the reaction energies and the binding energies.

The collapsed cycle, and therefore the convention adopted in **Fig. 6**, clearly do not reflect a physical reality (E+R and E•R are not at the same energy level; the real difference between E+P and E•P is ΔG^bind^_P_, not ΔG^bind^_P_ - ΔG^bind^_R_), but it is particularly convenient because it shows at a glance that the difference between the reaction energies in solution and in the enzyme is equal to the difference in binding energies for the enzyme between reactant and product. Thus, if we are only interested in relative binding energies the information already available from the QM/MM simulations of the reactions in solution and in the enzyme suffices to determine these energies.

Thus, based on our choice of origin, if the enzyme reaction profile ends below the solution profile (ΔΔG^bind^_P-R_ = ΔG^bind^_P_ - ΔG^bind^_R_<0) it means that the product (protonated bicyclic compound) binds more tightly to the enzyme than the reactant (protonated hydrolyzed biapenem). Conversely, if the enzyme reaction profile ends above the solution profile (ΔΔG^bind^_P-R_ = ΔG^bind^_P_ - ΔG^bind^_R_>0) it means that the product binds less tightly to the enzyme than the reactant. We also recall here that, since we are using a collapsed thermodynamic cycle (**Fig. 7C**) and the origins of the solution and enzyme profiles are not on an absolute scale (we only know the relative binding energies of reactant and product) (**Fig. 6A**), we can only evaluate the contribution of the various reaction steps to *k*_cat_, but not to *k*_cat_/*K*_m_.

Under enzyme configuration 1 (Green line in **Fig. 6A**) there is a single TS and the reaction barrier (22.4 kcal/mol) is slightly bigger than that calculated for the uncatalyzed reaction in solution (with *k*_1_≅*k*′≅0.00023 s^−1^, **Table 2**). Configuration 2 is similar to configuration 1 except that a hydroxide ion replaces the water near the Zn ion. The free energy profile calculated with this configuration (Magenta line in **Fig. 6A**) is more complicated because, as the reaction is driven toward the bicyclic compound, the proton on N4 is transferred to the hydroxide ion. Thus, although the simulation starts with protonated reactant, from the first TS onward the energy profile reflects the cyclization of unprotonated hydrolyzed biapenem. In this configuration of the enzyme a single TS (labeled TS2/3 in **Fig. 6A**) was identified in the RS N4⇒PS N4 step. The calculated energy barrier of 21.2 kcal/mol for this step (corresponding to a rate constant *k* = ∼0.0017 s^−1^) is slightly smaller than that of configuration 1. Simulation of the reaction time-course using the rate constants (**Table 2**) derived from the free energy differences shown in **Fig. 6A**, reveals that, if the reaction starts with 100% hydrolyzed biapenem deprotonated at N4 (RS N4), a transient chemical equilibrium is reached almost instantly, in which 3% of hydrolyzed biapenem is protonated at N4 (**Fig. 6B**); a stable chemical equilibrium with zero net fluxes, in which 32% of biapenem is in bicyclic form with N4 ionized (PS N4) and 68% in bicyclic form with N4 protonated (PS NH4), is reached within 10^4^ s. Thus, this reaction path is associated with the formation of a very stable anionic intermediate. However, since the rate at which the chemical equilibrium is reached is very slow (*k*′≅0.0016 s^−1^), this path cannot account for the formation of the bicyclic compound at a rate comparable with that observed for the enzymatic inactivation of biapenem (∼300 s^−1^) [19].

**Table 2:** Rate constants (s^−1^) for individual steps in the cyclization reaction of hydrolyzed biapenem in solution and in the configurations of the enzyme listed in **Table 1**.

	*k*_1_	*k_−_*_1_	*k*_2_	*k_−_*_2_	*k*_3_	*k_−_*_3_	*k*_4_	*k_−_*_4_
NH4 sol.	0.0023	5.2×10^−11^						
N4 sol.	1.0×10^−11^	6.1×10^10^	1.7×10^9^	5.7×10^11^	7.1×10^4^	1.6×10^−5^	2.2×10^12^	4.2×10^10^
NH4 enz. 1	2.3×10^−4^	2.2×10^−23^						
NH4 enz. 2	2.7×10^12^	8.4×10^10^	0.0017	2.8×10^−14^	3.8×10^9^	1.8×10^9^		
N4 enz. 3	1.1×10^3^	1.7×10^11^	2.3×10^4^	6.2×10^12^	9.6×10^8^	3.5×10^−13^	1.5×10^12^	6.38
N4 enz. 4	5.9×10^−7^	6.1×10^12^	4.3×10^5^	3.9×10^5^	1.0×10^9^	191.8	6.2×10^11^	1.1×10^−11^

In the next two configurations the reaction simulation started with hydrolyzed biapenem deprotonated at N4, but we also calculated the direct protonation step (RS N4 ⇒ RS NH4). The 3^rd^ configuration differs from the 1^st^ for the fact that Asp120 is protonated and donates a hydrogen bond to the nearby water, which in turn donates hydrogen bonds to biapenem N4 and to the δ-nitrogen of His118. In this configuration the reaction free energy profile (Black line with yellow circles in **Fig. 6A**) is somewhat similar to that of configuration 2, but the barrier between RS N4 and PS N4 is smaller (∼16.7 kcal/mol, corresponding to a rate constant *k* = 3.6 s^−1^). Simulation of the reaction time course by solving the ODEs of the system shows that, if the reaction starts with 100% hydrolyzed biapenem deprotonated at N4 (RS N4), a transient chemical equilibrium with almost zero net fluxes is reached very rapidly (*k* = ∼10^11^ s^−1^) at which essentially 100% of biapenem is hydrolyzed with N4 protonated. As expected from the overall negative ΔG^0^ value, very slow (*k*′≅10^−8^ s^−1^) complete conversion of this compound to the bicyclic compound with N4 protonated occurs over a time scale of 10^9^ seconds without any stable intermediates.

In the final configuration Asp120 and His196 are both protonated and donate a hydrogen bond to the water near the Zn ion; this water in return donates hydrogen bonds to biapenem N4 and to the δ-nitrogen of His118. In this configuration (Cyan line in **Fig. 6A**), the rate-limiting step for the cyclization reaction starting from hydrolyzed biapenem with N4 ionized is the proton transfer from the hydroxyethyl group to C2 (TS2, **Fig. 6A**). Although the overall reaction barrier from RS N4 to PS N4 (ΔG^‡^ = 9.8 kcal/mol, corresponding to a rate constant *k* = ∼4.0×10^5^ s^−1^, **Table 2**) is much smaller than in the uncatalyzed reaction in solution (red trace in **Fig. 6A**), direct conversion from RS N4 to RS NH4 is essentially barrierless, and this event is much favored with respect to the cyclization reaction. Simulation of the time course of the reaction shows a behavior very similar to that observed for enzyme configuration No. 3. For example, if the reaction starts with 100% hydrolyzed biapenem with N4 ionized (RS N4) this compound is almost instantly protonated at N4, and then slowly converted to the bicyclic compound over a time scale of ∼10^14^ seconds without any visible intermediates.

Changes in the entropic contributions (−T*S) to the free energy profiles as the reaction progresses toward the product(s) are shown in the lower quadrant of **Fig. 6A** (see also **Fig. 4A** for the corresponding entropic changes in solution). In agreement with earlier studies of the entropic effects in enzymatic reactions [35], [36], [37], [38], [39], [40], [41] these entropy changes do not exceed 3 kcal/mol, and are of the same magnitude for the reactions occurring in solution and in the enzyme active site.

The free energy profiles calculated for the post-hydrolysis reactions catalyzed under several configurations of the enzyme (although these were certainly not exhaustive of all the possible ionization states of the active site) suggest that the enzymatic formation of the bicyclic derivative of biapenem cannot occur at a rate comparable to the inactivation of biapenem by CphA measured experimentally (300 s^−1^) [19]. If the cleavage of the β-lactam ring leaves N4 ionized (negatively charged), a rotation of the hydroxyethyl moiety would bring the ensemble to a state (RS N4 in **Fig. 6A**) in which direct protonation of N4 appears to be kinetically favored (with the exception of configuration No. 2, **Fig. 6B**) with respect to the formation of the bicyclic compound. Altogether our simulations confirm the results of the calculations carried out by Wu *et al.* [28] with one enzyme configuration. Comparison of the free energy profiles in solution and in the enzyme suggests that (with the exception of configuration 2) hydrolyzed biapenem with N4 deprotonated binds less tightly to the enzyme than its protonated form (the RS N4 points of the enzyme profiles are above the solution profile, **Fig. 6A**), suggesting that RS N4 is the form released in solution. In all configurations the bicyclic compound (PS NH4) binds more tightly to the enzyme than the open ring form of biapenem (RS NH4): in configuration 2 the protonated and unprotonated form of the bicyclic compound have similar affinity for the enzyme.

### Metadynamics of the hydroxyethyl group rotation

In the original report on the formation of the bicyclic derivative of biapenem by CphA [19], it was assumed that the rotations of the C6 carboxylate and hydroxyethyl groups required to position optimally the hydroxyl moiety for the expected proton transfer to C2 would occur unhindered. Later work showed that these rotations are facilitated [21], and most recently Wu *et al.* [28] determined a barrier for the rotations of ∼7–8 kcal/mol based on QM/MM simulations. In this study, we have calculated the free energy surface (FES) associated with the two rotations, both in solution and in the enzyme, under different ionization states of the N4 nitrogen and of the C6 carboxylate by application of the metadynamics method [42]. Metadynamics is a technique in which the potential for one or more collective variables (CVs) is modified by periodically adding a repulsive potential of Gaussian shape at the location given by particular values of the variables. These repulsive Gaussians eventually fill up the well that is being sampled, and force the calculation to sample elsewhere. At certain points in the simulation, the sum of the Gaussians and the free-energy surface (FES) becomes flat, and the sum of the Gaussians provides the negative image of the FES. In our simulations the collective variables were the dihedral angle defined by atoms N4-C5-C6-C61 (see **Fig. 2**) of hydrolyzed biapenem or “Dihedral CV1”, and the dihedral angle defined by atoms C5-C6-C61-O62 of hydrolyzed biapenem or “Dihedral CV2”. With regard to this method, it is worth pointing out that while the history of the collective variables determines where the repulsive potentials are added, the entire protein and/or solvent keep moving during the metadynamics simulation, and thus are constantly reorganizing in response to changes of the two dihedral angles. As a consequence, the final free energy profile includes also the contribution from the reorganization energy of the protein and solvent.

**Figure 8:**
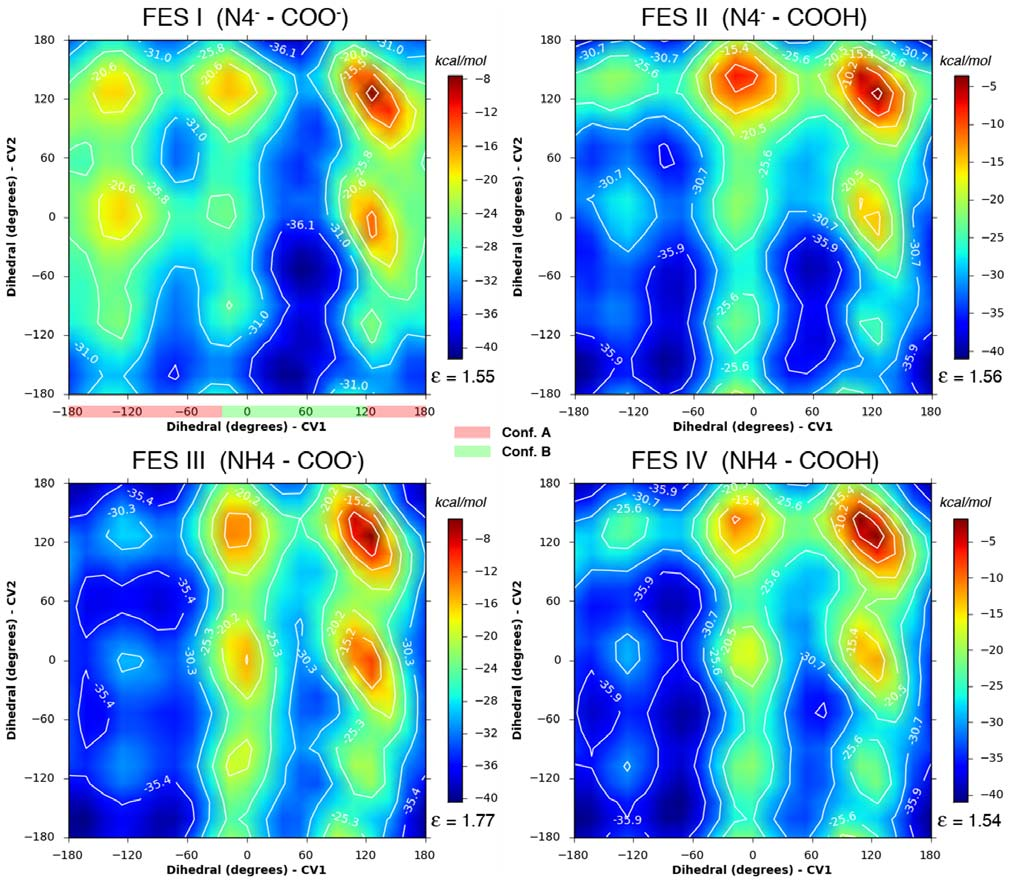
Free energy surfaces (FESs) of the hydroxyethyl group rotations in solution. FESs were calculated under four conditions corresponding to 1) deprotonated N4 and C6 carboxylate (FES I), 2) deprotonated N4 and protonated C6 carboxylate (FES II), 3) protonated N4 and deprotonated C6 carboxylate (FES III), 4) protonated N4 and C6 carboxylate (FES IV). The collective variables (CV) sampled in the metadynamics simulations were the dihedral angle defined by atoms N4-C5-C6-C61 (see Fig. 2) or “Dihedral CV1”, and the dihedral angle defined by atoms C5-C6-C61-O62 or “Dihedral CV2”. The ranges of Dihedral 1 values corresponding to Conformation A and Conformation B are highlighted in red and green, respectively, in the FES I panel. The start point for all simulations was conformation B. The average error ε (kcal/mol) of the FES, as calculated from equation (4) (see Methods) is shown in each panel below the color bar.

In our particular case, in order to verify that the results obtained were not dependent on the initial configuration, the metadynamics simulations were started from two different values of both CVs corresponding respectively to a conformation that does not allow the cyclization of hydrolyzed biapenem (Conformation A, see **Fig. 2A**), and a conformation that instead favors the cyclization (Conformation B, see **Fig. 2B**). Furthermore, two separate sets of simulations were carried out with hydrolyzed biapenem in solution or bound to the enzyme, respectively. Combined results from all the metadynamics simulations (starting from either conformation) are reported in **Table 3**. Partial results for the metadynamics starting from conformation A or B are reported in **Table S1**. As an example, we describe here in detail only the simulations that started from the conformation favoring cyclization (Conformation B) (**Figs. 8** **,** **9**). The FESs obtained from the simulations that started with conformation A are shown in **Figs. S4, S5.**

**Figure 9:**
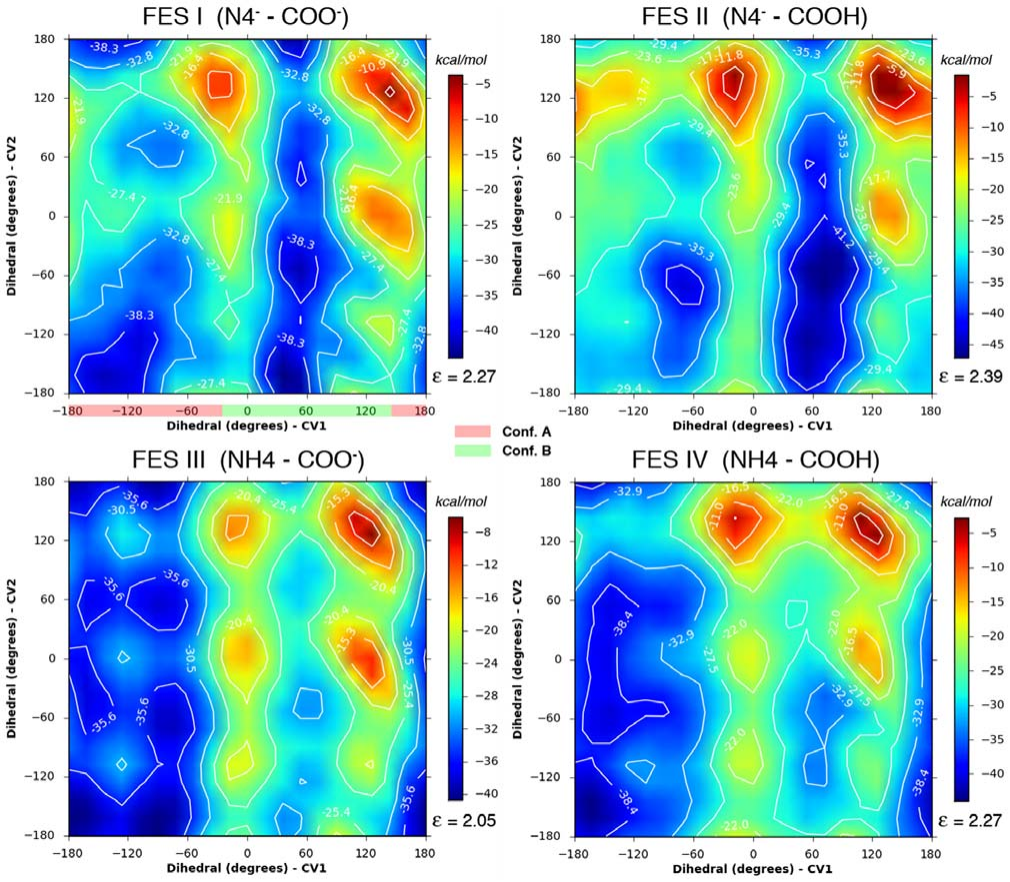
Free energy surfaces (FESs) of the hydroxyethyl group rotations in the enzyme. FESs for the hydroxyethyl group rotations occurring with hydrolyzed biapenem in the active site of CphA in the configuration No. 4 of **Table 1** were calculated under the same conditions and for the same collective variables as in **Fig. 8**. The start point for all simulations was conformation B. The start point for all simulations was conformation B. The average error ε (kcal/mol) of the FES, as calculated from equation (4) (see Methods) is shown in each panel below the color bar.

**Table 3:** Energetics of the rotation of the hydroxyethyl group of hydrolyzed biapenem in solution and in the enzyme (Configuration No. 4 in **Table 1** and **Fig. 6A**).

		ΔG^‡^(A→B)	ΔG^‡^(B→A)	ΔG^0^(A→B)
Free in solution	N4^−^-COO^−^	3.5 (1.2)	8.2 (0.9)	**−4.7** (0.4)
	N4^−^-COOH	11.6 (1.5)	9.8 (1.2)	1.8 (0.3)
	NH4-COO^−^	15.1 (0.9)	7.7 (0.2)	7.4 (1.1)
	NH4-COOH	15.3 (2.3)	10.3 (1.6)	5.0 (0.7)
Bound to the enzyme	N4^−^-COO^−^	12.0 (0.8)	14.3 (1.1)	**−2.3** (0.3)
	N4^−^-COOH	12.5 (2.2)	17.7 (0.2)	**−5.2** (2.4)
	NH4-COO^−^	15.8 (2.4)	5.9 (1.3)	9.9 (1.1)
	NH4-COOH	15.5 (1.4)	8.2 (1.2)	7.2 (2.6)

ΔG values (kcal/mol) are the mean and standard deviation (in parenthesis) of two metadynamics simulations started respectively from conformation A (**Fig. 2A**) or B (**Fig. 2B**) of hydrolyzed biapenem. Values in bold highlight reactions that occur spontaneously.

The FESs for the rotations occurring with hydrolyzed biapenem in solution were calculated under four different conditions. Under condition 1 (N4 and C6 carboxylate both deprotonated, **Fig. 8** **, FES I**), the global minimum on the FES (−41 kcal/mol, dihedrals 1 and 2 around 60° and −50°) corresponds to conformation B, favoring the formation of the bicyclic compound. The other two deep minima with dihedral 1 between 0° and 100° and dihedral 2 between −100° and ±180/+150° correspond to conformations that are separated from the most stable conformation by very small barriers, and thus can be collectively considered as part of conformation B. Two other more shallow minima (−36 kcal/mol, dihedral 1 around −75° or ±180° and dihedral 2 around −155°) correspond to conformations that might ensue immediately after the cleavage of the β-lactam ring and can be defined collectively as part of conformation A. Change from A to B (the rotation of biapenem hydroxyethyl moiety required for the cyclization to occur) is expected to be spontaneous under standard conditions with a barrier of ∼4 kcal/mol. Under condition 2 (deprotonated N4 and protonated C6 carboxylate, **Fig. 8** **, FES II**) conformation A is slightly more stable than conformation B, and the barrier between the two conformations is slightly higher (∼9–10 kcal/mol in both directions). Under condition 3 (protonated N4 and deprotonated C6 carboxylate, **Fig. 8** **, FES III**) the global minimum (at −40 kcal/mol) corresponds to conformation A; conformation B is less stable (−34 kcal/mol) and its conversion to conformation A occurs spontaneously over a barrier of ∼8 kcal/mol on the minimum energy path (MEP). Under condition 4 (N4 and C6 carboxylate both protonated, **Fig. 8** **, FES IV**) conformation A (−41 kcal/mol) is more stable than conformation B (−36 kcal/mol), and conversion of the latter to the former over a barrier of ∼9 kcal/mol is favored. Altogether the FESs suggest that in solution the conformation of hydrolyzed biapenem that leads to the formation of the bicyclic compound (conformation B) is unlikely to occur under any conditions (3 and 4) in which N4 is protonated (**Table 3**).

The FESs for the rotations occurring with hydrolyzed biapenem were calculated also for the compound bound to the configuration No. 4 in **Table 1** (Cyan trace in **Fig. 6A**), which was found to have the lowest barrier for the cyclization step (RS N4⇒PS N4). Also in this case, four different ionization states of hydrolyzed biapenem were studied (**Fig. 9**).

Under condition 1 (N4 and C6 carboxylate both deprotonated, **Fig. 9** **, FES I**), the global minimum (−44 kcal/mol) on the FES (dihedrals 1 and 2 around 45° and −160°) corresponds to a conformation very close to that which favors the formation of the bicyclic compound (Conformation B). Conformation A is less stable (at −42 kcal/mol); switch from A to B is favored over a barrier on the MEP of ∼12 kcal/mol. Under condition 2 (deprotonated N4, protonated C6 carboxylate, **Fig. 9** **, FES II**) conformation B (global minimum at −47 kcal/mol) is more stable than conformation A (local minimum at −44 kcal/mol); conversion from A to B is favored over a barrier of ∼14 kcal/mol on the MEP. Under condition 3 (protonated N4, deprotonated C6 carboxylate, **Fig. 9** **, FES III**) the global minimum (−41 kcal/mol) corresponds to conformation A and conformation B (at −32 kcal/mol) is less stable; switch from B to A is strongly favored (ΔG^0^ = −9 kcal/mol) over a barrier of only ∼5 kcal/mol. Under condition 4 (N4 and C6 carboxylate both protonated, **Fig. 9** **, FES IV**) conformation A (at −44 kcal/mol) is more stable than B (at −35 kcal/mol); conversion from B to A is favored (ΔG^0^ = −9 kcal/mol) over a barrier of only ∼7 kcal/mol on the MEP.

Altogether the FESs suggest that in the configuration of the enzyme considered in the metadynamics simulations (No. 4 in **Table 1**), if N4 of hydrolyzed biapenem is protonated conformation A is very stable and will not change to B spontaneously (**Table 3**). Conversely, if at the end of the hydrolysis reaction biapenem N4 is ionized (state labeled RS N4 in the free energy profiles of **Fig. 6A**), a switch from conformation A to B will occur spontaneously at rate comparable to that of the hydrolysis step (barrier of ∼12 kcal/mol) (**Table 3**).

## Discussion

The main obstacle to a clear understanding of the mechanism of metallo β-lactamases has been the absence of X-ray structures that show the exact position of the antibiotic in the reactant state (the Michaelis complex). For example, in the case of the B2 enzyme CphA from *Aeromonas hydrophila*, only the X-ray structure of the enzyme in complex with product is available [19]. Furthermore, this product is not the expected hydrolyzed β-lactam, but some further rearrangement of the molecule to a bicyclic compound. The only way this compound can form is if after the hydrolysis step a rotation of the C6 carboxylate and hydroxyethyl moieties brings the latter in a favorable position for proton transfer from the hydroxyl group to C2 and for attack of the hydroxyl oxygen onto C3 of the 5-membered ring. However, the experimental observation of the bicyclic derivative of biapenem bound to CphA is not itself evidence that this compound is formed inside the enzyme active site: hydrolyzed biapenem might be released from the enzyme, cyclization could occur in solution, and the bicyclic compound could bind back to the enzyme. For this reason, in analyzing the post-hydrolysis steps of biapenem inactivation we have simulated the reaction both in solution and in the enzyme. Free energy profiles were derived either from metadynamics under conditions of fixed chemical topology (rotations only), or from high-resolution QM/MM relaxed scans at the DFT/B3LYP level of theory for the chemical topologies that vary along the reaction coordinate (bond breaking/forming). We reiterate that at each point in the metadynamics or in the QM/MM scans both protein and/or solvent were allowed to move around the changing antibiotic, and thus the free energy profiles derived from the simulations reflect also the reorganization energy of the ensemble. One clear outcome of the calculations is that the energy landscape of the post-hydrolysis events is quite different depending on the protonation state of N4 in the 5-membered ring of hydrolyzed biapenem.

In solution, if N4 and the C6 carboxylate are deprotonated the hydroxyethyl group of hydrolyzed biapenem will rotate spontaneously (ΔG^0^ around −5 kcal/mol, **Table 3**) to reach a position compatible with the cyclization reaction. Once this position is reached, the following cyclization is highly favored (ΔG^0^ around −12 kcal/mol; *K*_eq_ = ∼10^10^), and will occur with a maximal rate of ∼130 s^−1^. Thus, if biapenem is released from the enzyme with N4 deprotonated, formation of the bicyclic compound is expected to occur in solution at an uncatalyzed rate comparable with the enzymatic hydrolysis (300 s^−1^) [19] of biapenem by CphA. Conversely, if biapenem is released from the enzyme with N4 protonated, rotation of the hydroxyethyl group in solution is not favored (ΔG^0^≥5 kcal/mol, **Table 3**), and the following cyclization reaction can only occur at a rate of at most ∼0.002 s^−1^. The shift of the equilibrium in favor of conformation B when N4 is deprotonated occurs also if hydrolyzed biapenem is bound to the enzyme, and is particularly pronounced if the C6 carboxylate is protonated (ΔG^0^ = −5.2 kcal/mol, **Table 3**), as expected to be at the end of the hydrolysis reaction (see **Fig. 2A**).

Our calculations suggest that formation of the bicyclic compound essentially does not occur inside the enzyme active site (**Fig. 6A**), but can occur in solution at a significant rate if hydrolyzed biapenem is released from the enzyme in a conformation in which the hydroxyethyl group is rotated (as in **Fig. 2B**). Under these conditions, the existence of a low energy path that involves a transient deprotonation of N4, would pull the equilibrium toward the formation of the bicyclic compound. On this basis we propose a model for the final steps of biapenem inactivation by CphA in which: 1) cleavage of the β-lactam ring leaves N4 ionized, 2) the hydroxyethyl moiety rotates to a position (state RS N4 in **Fig. 6A**) that allows the cyclization reaction, 3) hydrolyzed biapenem (with N4 ionized and the hydroxyethyl group rotated) is released from the enzyme (state RS N4 in **Fig. 4A**), 4) the cyclization reaction occurs in solution and leads to bicyclic biapenem with N4 protonated (PS NH4 in **Fig. 4A**), 5) the N4-protonated bicyclic compound binds back to the enzyme (**Fig. 2C**, PS NH4 in **Fig. 6A**) acting as an apparent product inhibitor [29]. It is worth noting that this model is consistent with the long known fact that in solution the open-ring form of β-lactams is a strong base that at equilibrium is mostly protonated at N4 [12], [14], [30], [31]. Our simulations only indicate that, from the kinetic point of view, the equilibrium corresponding to the bicyclic protonated derivative of carbapenems is reached through a path in which N4 is transiently ionized (**Fig. 4**).

This is a very important result because the reactant state of the cyclization reaction is the product state of the hydrolysis reaction, and identification of the correct mechanism for the former points also to the correct mechanism for the latter. For example, our calculations are consistent with earlier predictions by Xu *et al.* [21], [22], and with the most recent work by Wu *et al.* [28], but suggest a possible revision of other proposed mechanisms [27] in which the cleavage of the β-lactam ring of biapenem by CphA is associated with or immediately followed by protonation of N4 (which would prevent the rotation of the hydroxyethyl group to the conformation that favors cyclization). Our results also confirms the existence of a common mechanism for carbapenems hydrolysis in B2 and other types of MβLs [14], [15], [26], [43], in which ionized N4 is the leaving group, with the notable difference that protonation of N4 is not the main rate limiting step in B2 MβLs (**Fig. 6A**, see also [29]).

Finally, it is worth noting that the general mechanism proposed for the inactivation of β-lactams by MβLs does not offer much leeway for the design of inhibitors, as the most effective avenue to block the reaction remains the extraction or coordination of the active site metal by compounds presenting chelating groups (thiols, carboxylates, etc.) combined with an aromatic group (e.g., biphenyl tetrazoles, cysteinyl peptides, mercaptocarboxylates, succinic acid derivatives, etc.) [7], [44]. These compounds are likely to have similar effects on other metallo enzymes, and to be toxic in various degrees. There is therefore much need for new independent leads in the design of effective inhibitors of MβLs. A comparison of the energy profile of biapenem cyclization reaction in solution and in the enzyme indicates that in all four configurations of the enzyme that were studied in detail the bicyclic compound with N4 protonated (PS NH4) binds more tightly to the enzyme (**Fig. 6A**) than hydrolyzed biapenem. This is probably the reason why only the bicyclic compound was observed bound in the crystal structure of CphA [19]. It may also be of importance the fact that the interaction of the bicyclic derivative of biapenem with CphA (**Fig. 1**) has some features in common (namely Zn coordination via the ring nitrogen and a carboxylate oxygen) with the binding mode of pyridine-2,4-dicarboxylate (2,4-PDCA), which is the most effective competitive inhibitor of CphA [45] (*K*_i_ = 4.3 µM) known to date. The earlier crystallographic studies by Garau *et al.* [19], the kinetic studies by Sharma *et al.* [29], the computational studies by Wu *et al.* [28], and the results of this study all suggest that the bicyclic derivative of biapenem or other carbapenems has significant affinity for B2 MβLs. Different substituents in the pyrrole and/or oxazine ring may further strengthen its binding to the enzyme, and thus it may be possible to obtain a new series of competitive inhibitors of B2 MβLs by modification of this lead compound.

## Methods

### QM/MM simulations

QM/MM simulations [46] of the enzymatic and non-enzymatic post-hydrolysis reactions of biapenem were carried out with Jaguar/Qsite (Jaguar, version 7.7, Schrodinger, LLC, New York, NY, 2010). For the simulations of the enzymatic reaction an entire molecule of CphA from *Aeromonas hydrophila* in complex with the bicyclic derivative of biapenem (as derived from the refined coordinates of the X-ray structure, PDB entry 1X8I) was solvated inside a cubic box of SPC [47] waters of 70 Å side, retaining all the original structural waters. For regions outside the active site, the most probable protonation state of histidines, and the optimal orientation and tautomeric states of arginines, glutamines, and histidines were determined using the Protein Preparation Wizard of the Schrodinger Suite, which optimizes the protein hydrogen bond network by means of a systematic, cluster-based approach. Results obtained with this protocol were consistent with those obtained by assuming pH = 7.0 and determining the protein p*K*_a_'s with PROPKA [48], [49], [50]. As for the ionization states of residues of the active site involved in the binding of the metal and the antibiotic, various combinations were tested in different QM/MM simulations (see Results Section). After an initial geometry optimization (RMS deviation of the relaxed structure from the original crystal structure <0.3 Å), the solvent and all hydrogen atoms in the protein and its ligands were equilibrated during 100 ps of molecular dynamics (MD) at 300 K in the NPT ensemble with periodic boundary conditions and SHAKE constraints [51] using the OPLS-AA force-field [52], [53]. At this point the ensemble was readied for the QM/MM simulations under stochastic boundary conditions by removing any waters farther than 26 Å from the N4 of biapenem (the center of the QM/MM system) or 6.0 Å from any other protein atom. Afterwards, atoms farther than 24 Å from the center were frozen, atoms between 22 and 24 Å from the center were subjected to a 25 kcal/mol harmonic restraint. The QM region consisted of up to 92 atoms including the entire biapenem, Asp120 (beyond CB), His118, His196, and His263 (beyond CB), Cys221 (beyond CA), Ans233 (beyond CB), Zn^2+^, and the water molecule hydrogen bonded to Asp120 and loosely coordinated to Zn^2+^. Hydrogen link atoms were placed at the boundaries between the QM and MM region. The QM region was treated by density functional theory (DFT) [54], [55] using the B3LYP functional [56] with the lacvp* basis set (with added “+” diffuse function only for the metal ion). In this basis set all atoms H through Ar are described with 6-31G*, while heavier atoms (e.g, Zn) are modeled using the LANL2DZ effective core potentials basis set. The MM region was represented with the 2005 OPLS-AA force-field.

With regard to the choice of quantum method, DFT is by far the most widely used one to describe electronic structures, with B3LYP [56], [57] being probably the most popular functional. In its basic form, B3LYP is a correlation-exchange functional, whose exchange component is partly the local Slater-Dirac exchange, partly the exact Hartree-Fock (HF) exchange (at 20% level), and partly the Becke 88 exchange functional [58]. Although B3LYP is widely used, the M06 suite of density functionals [59] is also considered a good alternative to B3LYP. For this reason, and as a mean to test the sensitivity of the calculated energies to the specific functional used, ΔH^‡^, ΔH^0^ and ΔG^‡^, ΔG^0^ values for some of the points in the solution profiles of **Fig. 4** and in the enzyme profile No. 4 of **Fig. 6** were calculated also using the M06 functional (**Table S2**): the largest differences in calculated energies between B3LYP and M06 did not exceed 2–3 kcal/mol.

Upon initial optimization of the quantum region in the QM/MM ensemble of CphA in complex with the bicyclic compound, we applied a dynamic constraint to transfer the H2 hydrogen from C2 to O62, causing the opening of the saturated oxazine ring. A second dynamic constrain was then applied to rotate the C6 hydroxyethyl and carboxylate moieties around the C5–C6 bond, such that O62 would come in close proximity of C2. This procedure generated the open-ring form of biapenem, whose geometry was further optimized by QM/MM, and which represents the reactant state (RS) of the reaction in our simulations.

The importance of comparing the enzymatic to the non-enzymatic reaction occurring inside a box of water, as opposed to using full DFT on a simpler truncated or model system in the gas-phase, has been stressed repeatedly [40], [41], [60]. For this reason, in a separate set of experiments, the open ring form of biapenem was immersed in a cubic box of 60 Å side of explicit SPC waters. After an initial geometry optimization, the solvent and all hydrogen atoms in biapenem were equilibrated with 100 ps of molecular dynamics (MD) at 300 K in the NPT ensemble with periodic boundary conditions and SHAKE constraints using the OPLS-AA force-field. For the QM/MM simulation, just biapenem and a single water molecule that could act as proton donor/acceptor to/from biapenem N4 were treated quantum mechanically by DFT(B3LYP) at the same level of theory used for the simulation of the enzymatic reaction. The MM region, which consisted of all the other water molecules inside a sphere of 26 Å radius around biapenem N4, was treated using the 2005 OPLS-AA force field. Water molecules outside this range were removed, resulting in a spherical ensemble. Water molecules between 23 and 26 Å from the sphere center were restrained harmonically.

QM/MM potential energy surfaces (PESs) were constructed with relaxed scans (full geometry optimization at each scan point) employing the C–H bond between H2^BIA^ and C2^BIA^, the O–C bond between O62^BIA^ and C3^BIA^, and the H–O and H–N bond between O^WAT^ and N4^BIA^ as reaction coordinates. Points were obtained for each PES by constraining the grid coordinates (at 0.05 Å intervals) and minimizing the energy with respect to the remaining parameters. Transition states (TSs) were refined by the quadratic synchronous transit method (QST) [61], [62], [63] utilizing two points of the PES on opposite sides of the TS, and confirmed to correspond to a single negative frequency. Total enthalpies (*H*) at 1 atm, 298.15 K for the RS, TS(s), and PS were calculated from the vibrational properties of the states as the sum of the total internal energy *U_tot_* (*U_tot_* = *QM/MM Energy*+*Zero Point Energy*+*U_trans_*+*U_rot_*+*U_vib_*) and the *pV* (pressure×volume) term. Total free energies (G) at 1 atm, 298.15 K were calculated also from the vibrational properties as *G* = *H* – T*S. A scaling factor of 0.9614 (as applicable to SCF calculations with B3LYP and 6-31G* [64]) and an inclusion threshold of 10.0 cm^−1^ were applied to vibrational frequencies prior to the calculation of thermochemical properties. Reaction and activation enthalpies (*ΔH^0^*, *ΔH* ^‡^) and free energies (*ΔG^0^*, *ΔG* ^‡^) were calculated as the differences *H* ^PS^-*H* ^RS^, *H* ^TS^-*H* ^RS^, *G* ^PS^-*G* ^RS^, *G* ^TS^-*G* ^RS^ between the total enthalpies and free energies at the various stationary and transition states.

With regard to the method used to calculate the free energies profiles from the QM/MM simulations, entropic contributions were derived from the vibrational properties of individual stationary point and TSs and not from a free-energy perturbation (FEP) [65] procedure, which would have been prohibitive at the level of theory (DFT-B3LYP) adopted in this study. For this reason, in the figures that show the free energy changes along the reaction in solution and in the enzyme (**Figs. 4** **,** **6**), we also show the corresponding entropy (−T*S) changes. This is because in several studies of the entropic effects in enzymatic reactions [35], [36], [37], [38], [39], [40], [41] it was found that most of the entropy changes occur during substrate binding, and that the remaining entropy changes as the reaction progresses toward the product(s) typically do not exceed 3 kcal/mol, and are not significantly different in the enzyme active site with respect to the same reaction in solution. In agreement with these studies we found that the entropy changes during the post-hydrolysis reactions of biapenem in solution and in the enzyme did not exceed 3 kcal/mol. The concurrence of these values with those found for several other types of reactions suggests that the free energy profiles determined in this study, while not derived from a full FEP analysis, are nonetheless reasonably accurate.

### Metadynamics

Free energy surfaces (FESs) for the rotation in solution and in the enzyme of the hydroxyethyl and carboxylate groups around the C5–C6 bond of hydrolyzed biapenem (this is the dihedral angle defined by the four atoms N4-C5-C6-C61, see **Fig. 2B**), and of the hydroxyethyl moiety alone around the C6–C61 bond (this is the dihedral angle defined by the four atoms C5-C6-C61-O62, see **Fig. 2B**) were calculated with a metadynamics simulation with Desmond v3.0 [66]. Metadynamics [42] is a type of free energy perturbation (FEP) [65] method, which enhances the sampling of the free energy space by biasing against previously visited values of some specified collective variables (CVs; in our case the two dihedral angles). The biasing is achieved by periodically dropping repulsive kernels of Gaussian shape at the current location of the simulation in the phase-space of the collective variables. The accumulation of the Gaussian potential encourages the system to explore new values of the collective variables, and allows the crossing of barriers much more quickly than it would occur in standard dynamics.

For the metadynamics simulation of hydrolyzed biapenem in solution the ensemble was constructed by adding explicit SPC water molecules (and appropriate counterions to achieve electroneutrality) inside an orthorhombic box leaving a minimum distance of 20 Å between any biapenem atom and the edge of the box. For the metadynamics simulation of biapenem bound to the enzyme the ensemble was similarly constructed with explicit water molecules and counterions leaving a minimum distance of 12 Å between any protein atom and the edge of the box. In both cases, the ensemble was subjected to energy minimization under periodic boundaries condition first with the steepest descent (SD) method until a gradient threshold of 25 kcal/mol/Å was achieved, and then with the LBFGS [67] until a convergence threshold of 1 kcal/mol/Å was met. The 2005 OPLS-AA force-field was used in all calculations. Short-range Coulombic interactions were calculated with a cutoff radius of 9.0 Å, while long range interactions were calculated with the smooth particle mesh Ewald (tolerance of 1e^−9^) [68]. For each condition tested, the metadynamics simulation was carried out for 21 ns in the NPT ensemble at 298.15K (25°C), using the Nose-Hoover thermostat method [69] (relaxation time of 1.0 ps), and the Martyna-Tobias-Klein barostat method [70] (isotropic coupling of the cell along all three axes to a reference pressure of 1.01325 atm and a relaxation time of 2 ps). Integration was carried out with the RESPA integrator [71] using time-steps of 2.0 fs, 2.0 fs, and 6.0 fs for the bonded, van der Waals, and short- and long-range electrostatic interactions. SHAKE constraints were imposed on all the heavy-atom-hydrogen covalent bonds. Coulombic interactions were calculated as for the minimization protocol. Coordinates were saved every 4.8 ps. Before the 21.0 ns productive run of the simulation, the ensemble was relaxed using the following protocol: 1) 12 ps in the NVT ensemble at 10 K with a fast relaxation constant and non-hydrogen solute atoms restrained; 2) 12 ps in the NPT ensemble at 10 K and 1 atm, with a fast temperature relaxation constant, a slow pressure relaxation constant, and non-hydrogen solute atoms restrained; 3) 24 ps at 298.15 K and 1 atm with other conditions as in step 2; 4) 24 ps at 298.15 K and 1 atm with a fast temperature relaxation constant and a fast pressure relaxation constant.

The parameters that control the accuracy of the metadynamics simulation are the height and width of the repulsive Gaussian potential and the interval at which the Gaussian potentials are added. In all the simulations the height was 0.1 kcal/mol, the width was 5 degrees, and the interval was 0.09 ps. The accuracy of the results increases as the time interval increases, and for a given interval it is inversely proportional to the height of the Gaussian potential. In order to obtain an accurate representation of the FES it is very important to verify that the metadynamic simulation has converged. However, in general it is difficult to decide when to terminate a metadynamics run, because in a single run the free energy does not converge to a definite value but fluctuates around the correct result. Reducing the bias potential deposition rate brings the microscopic variables closer to thermodynamic equilibrium, but increases the time required to fill the FES; the deposition interval we have used (0.09 ps) is a reasonable compromise between accuracy and speed. With respect to the length of the simulations, as a general rule, if one is interested in reconstructing the FES, the simulation should be stopped when the motion of the CVs becomes diffusive in the region of interest. One way to assess this is to verify that all second order saddle points are not flat at the top (free energy of 0), but have negative values of free energy (indicating that the CVs are diffusing over those points). An example of the progressive achievement of this diffusive motion for one of the conditions of **Fig. 9** as the simulation time progresses is shown in **Fig. S6**: all second order saddle points in the landscape assume negative values of energy already at 18 ns. Data reported in **Table 3** are derived from the FESs calculated at 21 ns (see also **Figs. 8** **–** **9**).

Determination of the uncertainty present in the metadynamics FES is also a difficult problem. In theory, in a single metadynamics run the average error *ε* is given by:
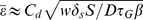
(3)where *C_d_* is a constant (≈0.3 for 2 CVs), *w*, δ_s_, and τ_G_ are the Gaussian height (kcal/mol), width (rad), and deposition interval (ps), *S* is the range of the CVs, *D* (rad^2^/ps) is the intrinsic system diffusion coefficient in the CV space, and *β* is (*k_B_T*)^−1^ [72], [73]. This means that the error of a metadynamics reconstruction is inversely proportional to the square root of the total simulation time, measured in units of the diffusion time. This error will be large for slowly diffusing systems, in which the walker takes a long time to explore the CV space. Alternatively, the average error can also be expressed as a function of the total simulation time as:
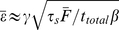
(4)where *τ_s_* = *S^2^/D*, *F* is the average depth of the energy wells in the landscape, and *γ*≈1.5 for 2 CVs. The theoretical errors calculated in this way are shown in each panel of **Figs. 8** **–** **9**. In practice the application of equation (4) is not straightforward, and the error in the free-energy profile is usually estimated by comparing independent runs [74], [75]. Since we were primarily interested in the conversion between the A and B conformations of the ligand, we have carried out a metadynamics simulation for each of the conditions shown in **Figs. 8** **–** **9** starting from either one of the two conformations. For this purpose, prior to the metadynamics run the dihedrals corresponding to the two CVs were manually altered such that they would fall onto different minima of the FES corresponding to these conformations. The system was then minimized and equilibrated prior to the productive run as described above. The progression of the metadynamics runs starting from the two initial conformations for one of the conditions of **Fig. 9** (N4^−^-COO^−^) is shown in **Fig. S6**. Although the two FESs are clearly different at 6 ns, they become progressively similar to each other as the simulation progresses.

The stability of the protein∶ligand complexes during the course of the metadynamics simulations was verified by recording the Cα-RMSD of CphA at different times with respect to time 0, and the distance between the active site Zn^+2^ ion and one of the oxygen atoms of the C3 carboxylate moiety of hydrolyzed biapenem. This analysis shows the protein to retain its native folding and the ligand to remain stably bound to the protein throughout the runs (**Fig. S7**) for the four conditions of **Fig. 9** examined by metadynamics.

### Kinetic Simulations

Steady states, time courses, and net rate constants *k*′(RS⇒PS) for the conversion of RS into PS (the apparent 1^st^ order rate constant for PS formation starting from 100% RS and 0% PS) were computed using a deterministic model with Copasi 4.6 [76]. In this approach, Copasi uses the ordinary differential equations (ODE) solver LSODA [77] to follow the time course defined by the differential equations that describe the model until a steady state is reached. For example, for the multistep reaction of cyclization in water or in the enzyme with 5 stationary points (SPs) and 4 TSs (**Figs. 4** **,** **6**) the ODEs describing the overall conversion from reactant to product are:

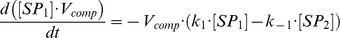

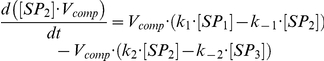

and so on until the last step:

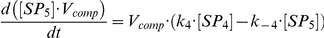

where V_comp_ is the volume of the compartment that is being simulated, and the rate constants are represented with the Cleland notation (e.g. *k*_1_ and *k*_−1_ are the forward and backward rate constants for the conversion of SP_1_ to SP_2_).

## Supporting Information

Figure S1
**Trigonal collapsed cycle with right apex.** **A.** Thermodynamic cycle relating the energy of the cyclization reaction in solution with the energy of the same reaction in the enzyme active site. E•R and E•P are the enzyme∶reactant and the enzyme∶product complexes, respectively. **B.** Intermediate step leading to the collapsed cycle shown in panel C. **C.** Collapsed cycle in which the same energy quantity (ΔG^bind^_P_) has been subtracted from the vertical legs of the cycle shown in panel A. The cycle gives origin to the following algebraic relationships:
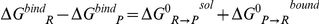


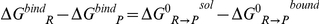

(TIF)

Figure S2
**Trigonal collapsed cycle with top apex.** **A.** Thermodynamic cycle relating the energy of the cyclization reaction in solution with the energy of the same reaction in the enzyme active site. E•R and E•P are the enzyme∶reactant and the enzyme∶product complexes, respectively. **B.** Intermediate step leading to the collapsed cycle shown in panel C. **C.** Collapsed cycle in which the same energy quantity (ΔG^0^_R→P_^sol^) has been subtracted from the horizontal branches of the cycle shown in panel A. The cycle gives origin to the following algebraic relationships:
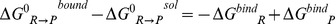


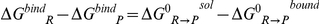

(TIF)

Figure S3
**Trigonal collapsed cycle with bottom apex.** **A.** Thermodynamic cycle relating the energy of the cyclization reaction in solution with the energy of the same reaction in the enzyme active site. E•R and E•P are the enzyme∶reactant and the enzyme∶product complexes, respectively. **B.** Intermediate step leading to the collapsed cycle shown in panel C. **C.** Collapsed cycle in which the same energy quantity (ΔG^0^_R**→**P_^bound^) has been subtracted from the horizontal branches of the cycle shown in panel A. The cycle gives origin to the following algebraic relationship:
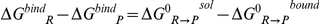

(TIF)

Figure S4 **Free energy surfaces (FESs) of the hydroxyethyl group rotations in solution.** The start point for all simulations was conformation A of hydrolyzed biapenem. FESs were calculated under four conditions corresponding to 1) deprotonated N4 and C6 carboxylate (FES I), 2) deprotonated N4 and protonated C6 carboxylate (FES II), 3) protonated N4 and deprotonated C6 carboxylate (FES III), 4) protonated N4 and C6 carboxylate (FES IV). The collective variables (CV) sampled in the metadynamics simulations were the dihedral angle defined by atoms N4-C5-C6-C61 (see Fig. 2) or “Dihedral CV1”, and the dihedral angle defined by atoms C5-C6-C61-O62 or “Dihedral CV2”. The ranges of Dihedral 1 values corresponding to Conformation A and Conformation B are highlighted in red and green, respectively, in the FES I panel.
(TIF)

Figure S5 **Free energy surfaces (FESs) of the hydroxyethyl group rotations in the enzyme.** The start point for all simulations was conformation A of hydrolyzed biapenem. FESs for the hydroxyethyl group rotations occurring with hydrolyzed biapenem in the active site of CphA in the configuration No. 4 of **Table 1** were calculated under the same conditions and for the same collective variables as in **Fig. S4**.
(TIF)

Figure S6 **Progression of the metadynamics FESs with time.** The progression of the. FESs for the condition corresponding to deprotonated N4 and deprotonated C6 carboxylate is shown for different simulation times. Panels on the left refer to the metadynamics simulation that started from Conformation A. Panels on the right refer to the metadynamics simulation that started from Conformation B. A black dot in the 6 ns panels shows the actual value of the CVs at the beginning of the simulations.
(TIF)

Figure S7 **Stability of CphA and of its complex with hydrolyzed biapenem during the metadynamics simulations.** In each panel, the blue trace represents the Cα-RMSD of CphA at different times with respect to time 0; the red trace is the distance between the active site Zn^+2^ ion and one of the oxygen atoms of the C3 carboxylate moiety of hydrolyzed biapenem. Panels A through D represent respectively the four conditions tested in metadynamics corresponding to 1) deprotonated N4 and C6 carboxylate (FES I of Fig. 9), 2) deprotonated N4 and protonated C6 carboxylate (FES II of Fig. 9), 3) protonated N4 and deprotonated C6 carboxylate (FES III of Fig. 9), 4) protonated N4 and C6 carboxylate (FES IV of Fig. 9).
(TIF)

Table S1 **Energetics of the rotation of the hydroxyethyl group of hydrolyzed biapenem.** Metadynamics of biapenem in solution and in the enzyme (Configuration No. 4 in **Table 1** and **Fig. 6A**) were carried out starting from conformation A (**Fig. 2A**) or B (**Fig. 2B**). ΔG values are in kcal/mol.
(DOC)

Table S2 **Sensitivity of the calculated energies to the functional used.** Some of the points of the energy profiles for the cyclization reaction of hydrolyzed biapenem in solution (**Fig. 4A**) and in the enzyme (Configuration No. 4 in **Table 1** and **Fig. 6A**) were calculated also using the M06 functional. Energies are in kcal/mol.
(DOC)
