# Supplementary figures and images for: Biapenem Inactivation by B2 Metallo β-Lactamases: Energy Landscape of the Post-Hydrolysis Reactions
Source: PLoS One. 2012 Jan 12;7(1):e30079. doi: 10.1371/journal.pone.0030079 (PMC3260057; doi:10.1371/journal.pone.0030079)

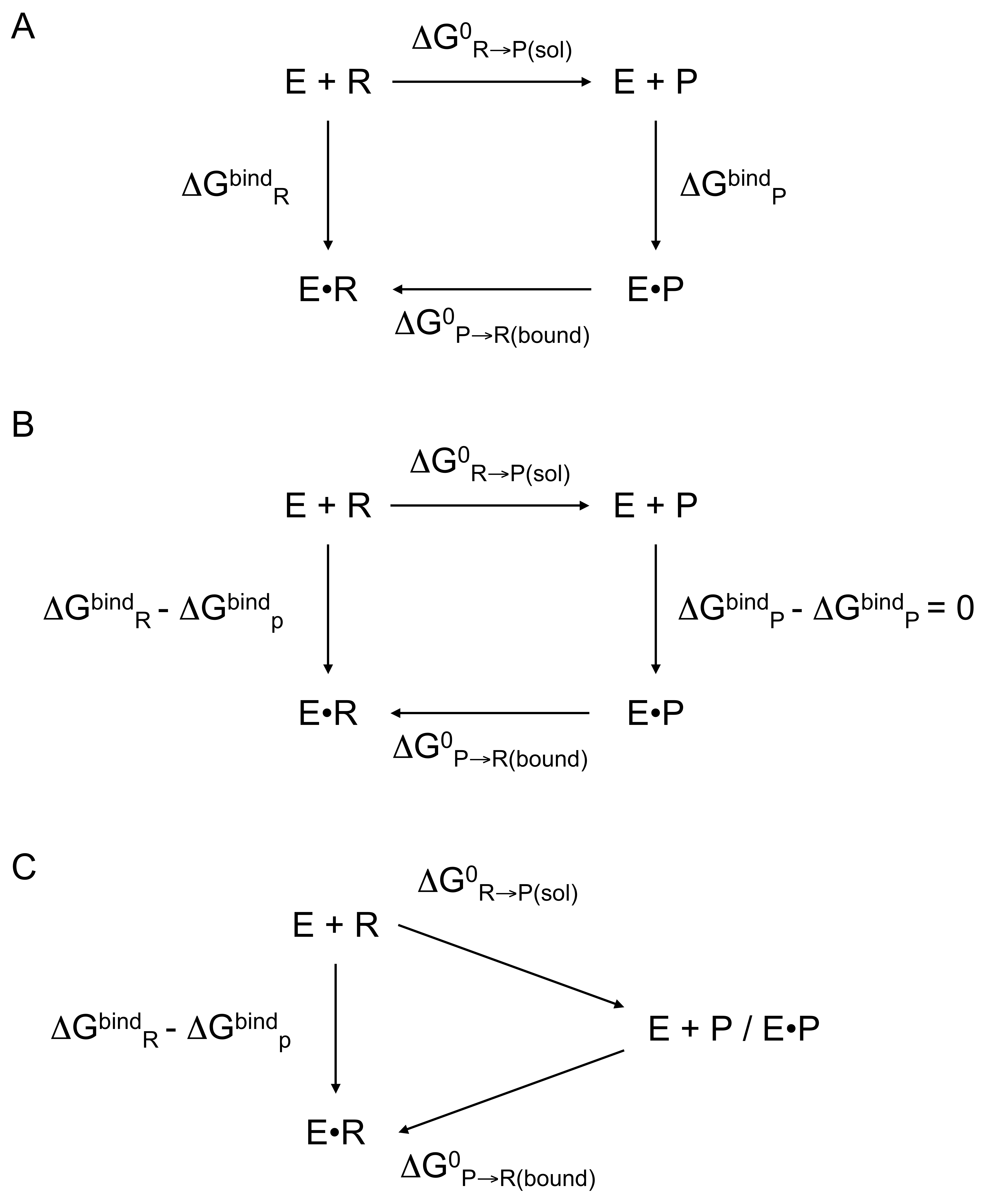

Supplement: Figure S1 — Trigonal collapsed cycle with right apex. A. Thermodynamic cycle relating the energy of the cyclization reaction in solution with the energy of the same reaction in the enzyme active site. E•R and E•P are the enzyme∶reactant and the enzyme∶product complexes, respectively. B. Intermediate step leading to the collapsed cycle shown in panel C. C. Collapsed cycle in which the same energy quantity (ΔGbind P) has been subtracted from the vertical legs of the cycle shown in panel A. The cycle gives origin to the following algebraic relationships: (TIF) [file pone.0030079.s001.tif]

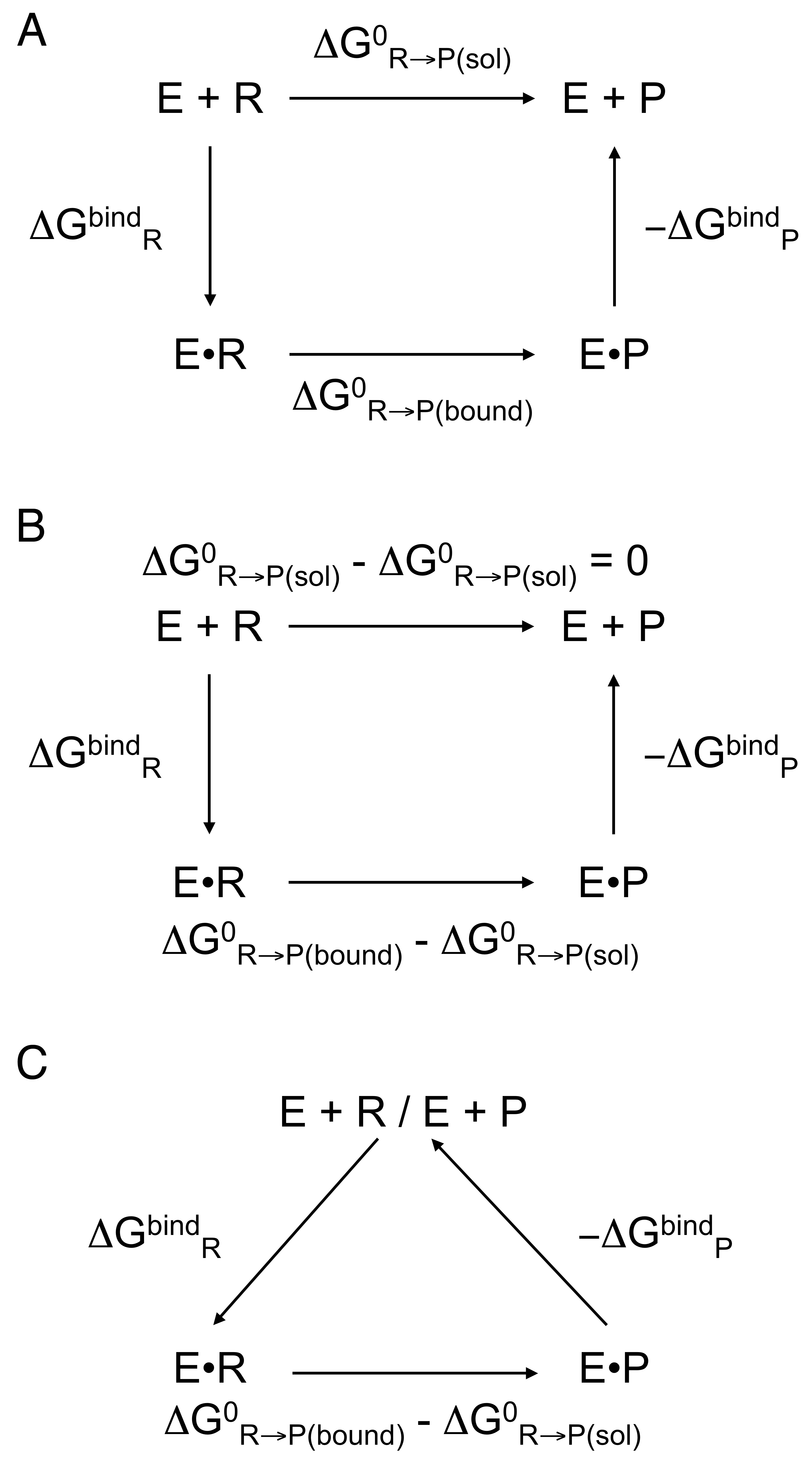

Supplement: Figure S2 — Trigonal collapsed cycle with top apex. A. Thermodynamic cycle relating the energy of the cyclization reaction in solution with the energy of the same reaction in the enzyme active site. E•R and E•P are the enzyme∶reactant and the enzyme∶product complexes, respectively. B. Intermediate step leading to the collapsed cycle shown in panel C. C. Collapsed cycle in which the same energy quantity (ΔG0 R→P sol) has been subtracted from the horizontal branches of the cycle shown in panel A. The cycle gives origin to the following algebraic relationships: (TIF) [file pone.0030079.s002.tif]

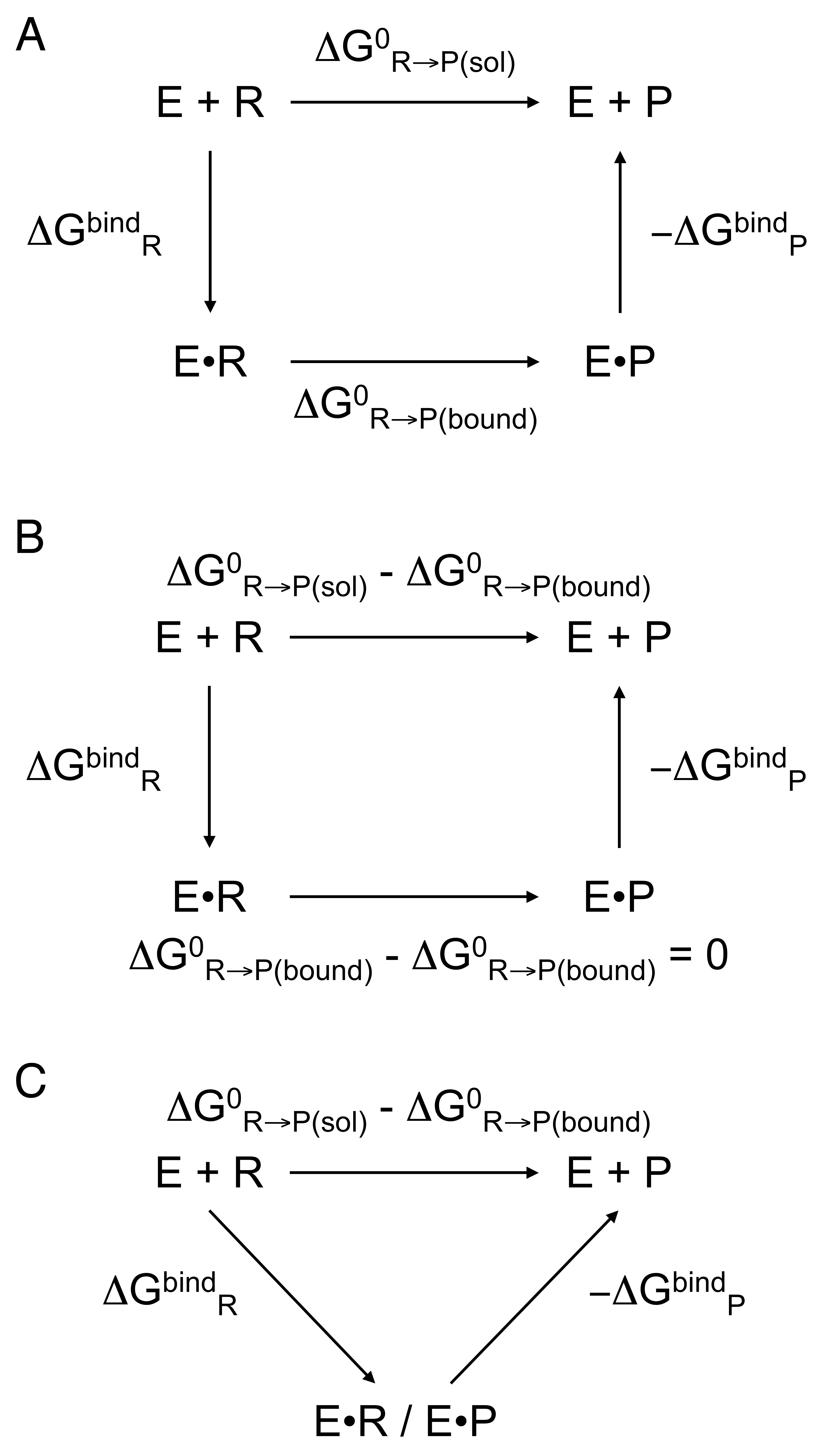

Supplement: Figure S3 — Trigonal collapsed cycle with bottom apex. A. Thermodynamic cycle relating the energy of the cyclization reaction in solution with the energy of the same reaction in the enzyme active site. E•R and E•P are the enzyme∶reactant and the enzyme∶product complexes, respectively. B. Intermediate step leading to the collapsed cycle shown in panel C. C. Collapsed cycle in which the same energy quantity (ΔG0 R→P bound) has been subtracted from the horizontal branches of the cycle shown in panel A. The cycle gives origin to the following algebraic relationship: (TIF) [file pone.0030079.s003.tif]

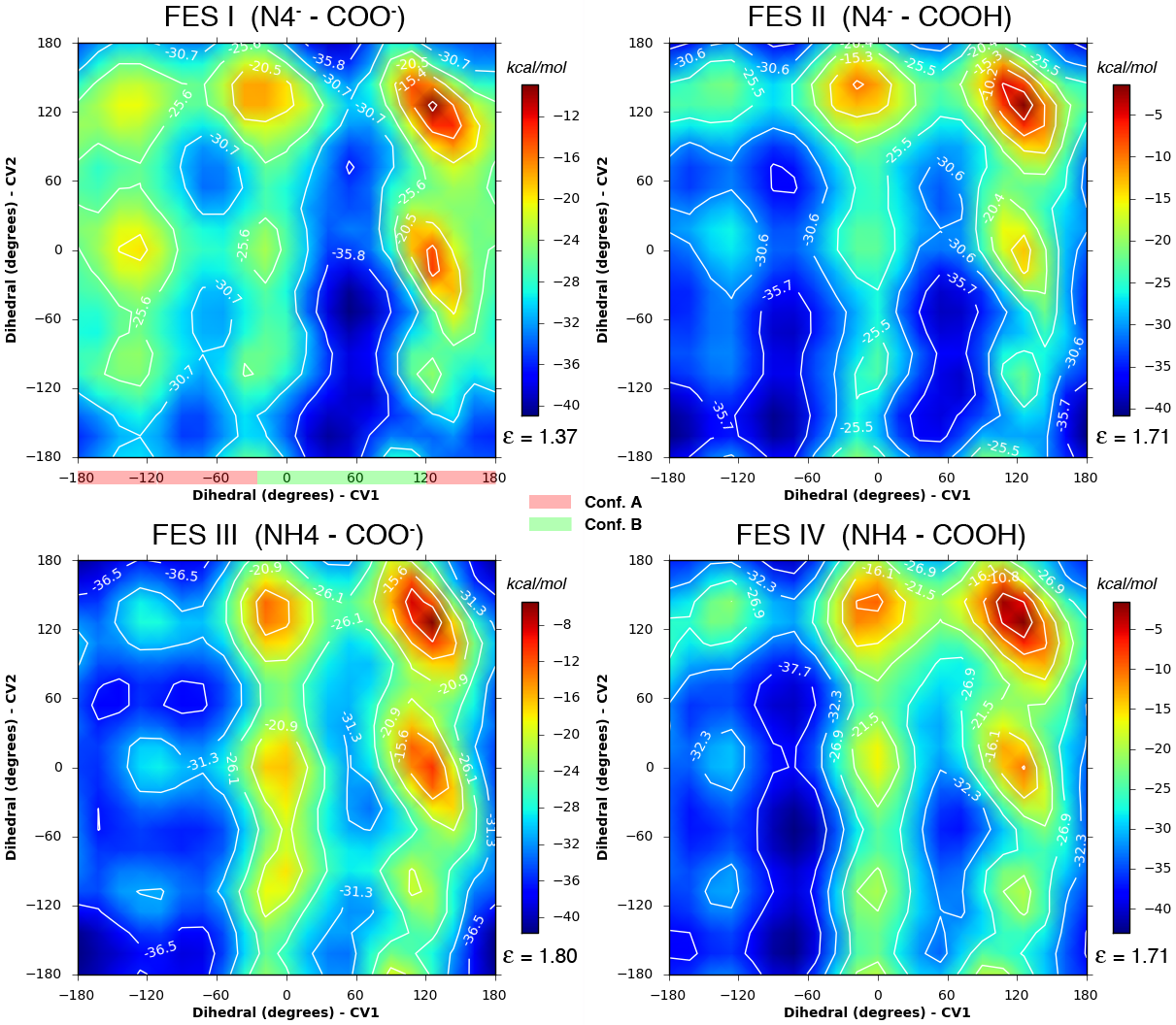

Supplement: Figure S4 — Free energy surfaces (FESs) of the hydroxyethyl group rotations in solution. The start point for all simulations was conformation A of hydrolyzed biapenem. FESs were calculated under four conditions corresponding to 1) deprotonated N4 and C6 carboxylate (FES I), 2) deprotonated N4 and protonated C6 carboxylate (FES II), 3) protonated N4 and deprotonated C6 carboxylate (FES III), 4) protonated N4 and C6 carboxylate (FES IV). The collective variables (CV) sampled in the metadynamics simulations were the dihedral angle defined by atoms N4-C5-C6-C61 (see Fig. 2) or “Dihedral CV1”, and the dihedral angle defined by atoms C5-C6-C61-O62 or “Dihedral CV2”. The ranges of Dihedral 1 values corresponding to Conformation A and Conformation B are highlighted in red and green, respectively, in the FES I panel. (TIF) [file pone.0030079.s004.tif]

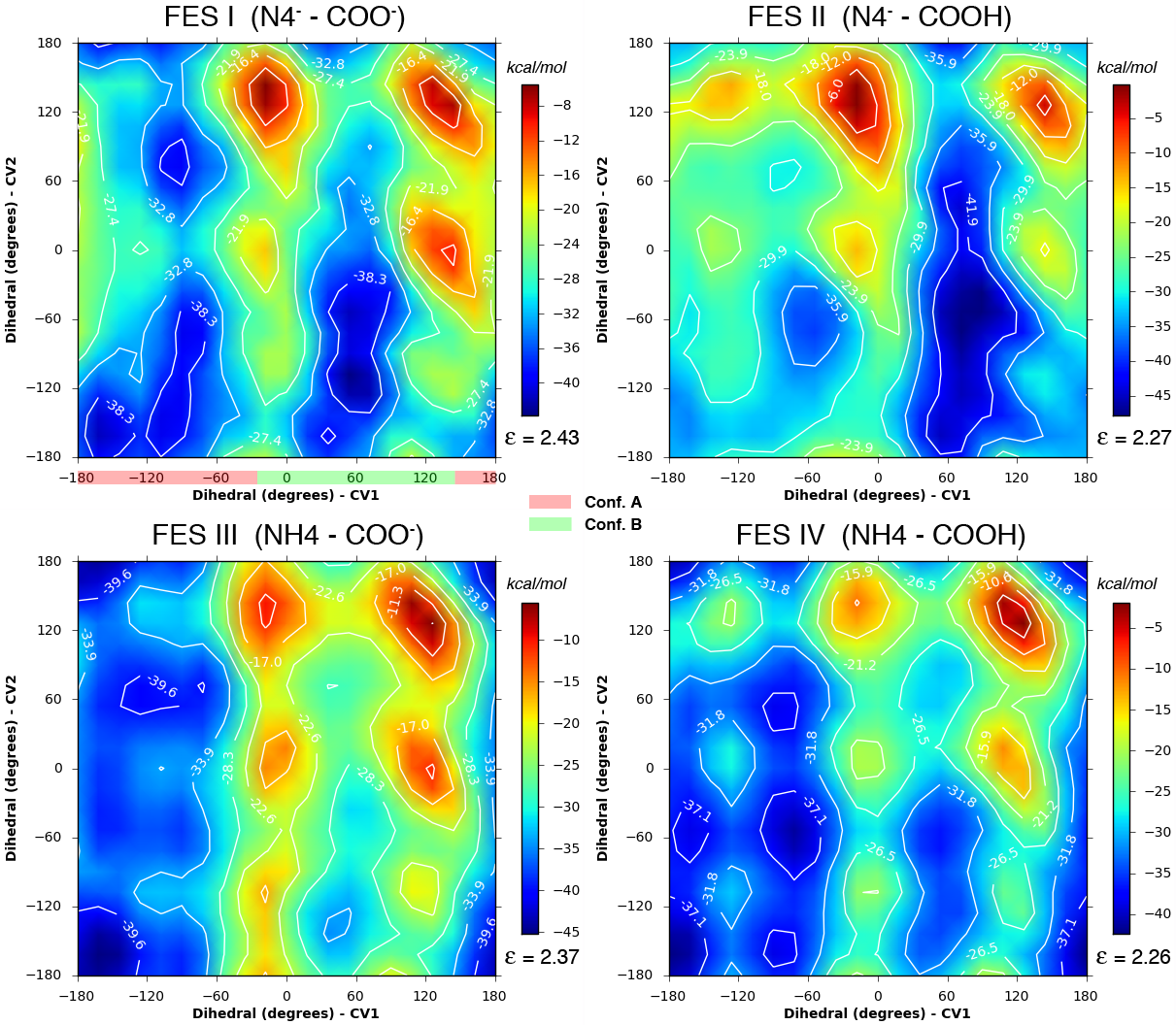

Supplement: Figure S5 — Free energy surfaces (FESs) of the hydroxyethyl group rotations in the enzyme. The start point for all simulations was conformation A of hydrolyzed biapenem. FESs for the hydroxyethyl group rotations occurring with hydrolyzed biapenem in the active site of CphA in the configuration No. 4 of Table 1 were calculated under the same conditions and for the same collective variables as in Fig. S4. (TIF) [file pone.0030079.s005.tif]

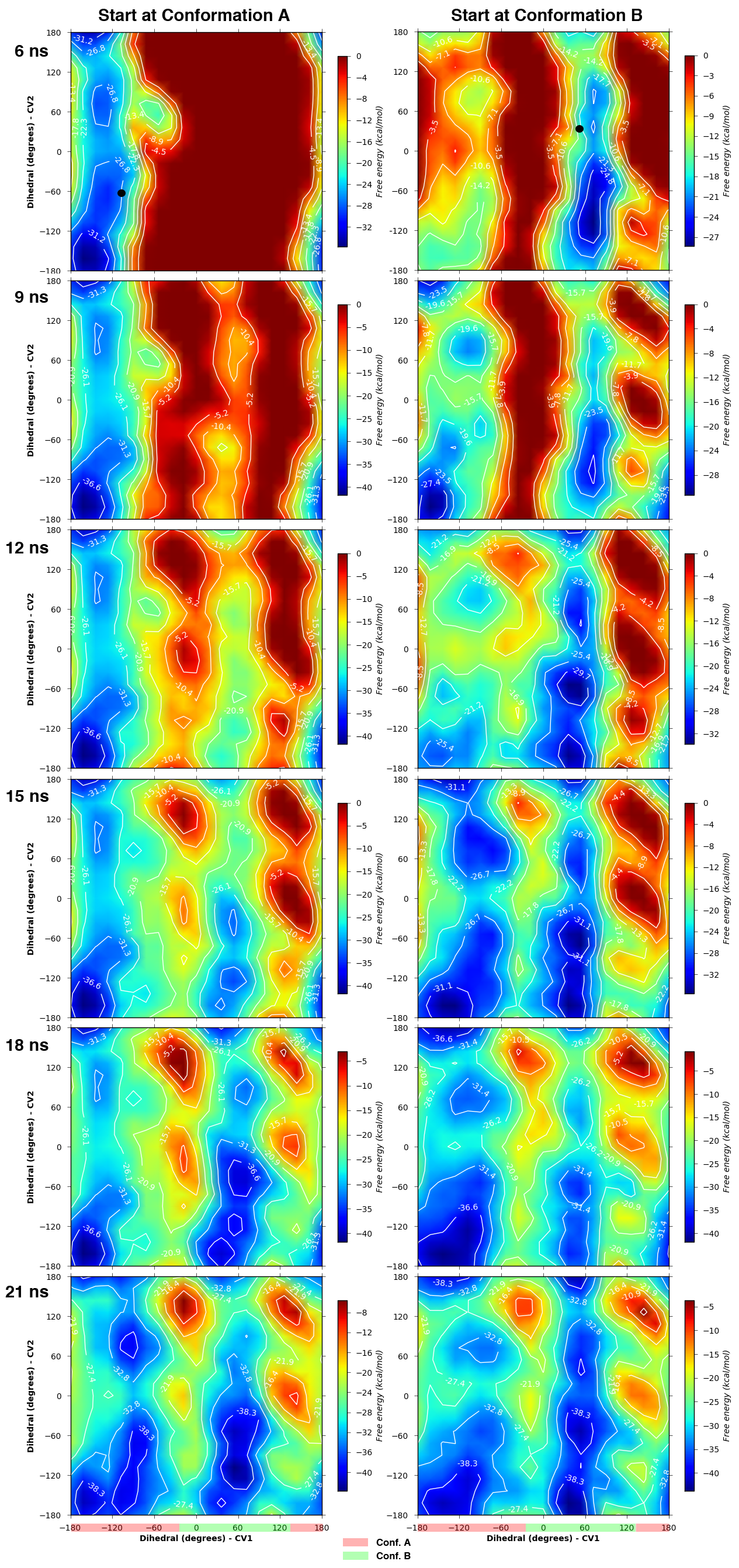

Supplement: Figure S6 — Progression of the metadynamics FESs with time. The progression of the. FESs for the condition corresponding to deprotonated N4 and deprotonated C6 carboxylate is shown for different simulation times. Panels on the left refer to the metadynamics simulation that started from Conformation A. Panels on the right refer to the metadynamics simulation that started from Conformation B. A black dot in the 6 ns panels shows the actual value of the CVs at the beginning of the simulations. (TIF) [file pone.0030079.s006.tif]

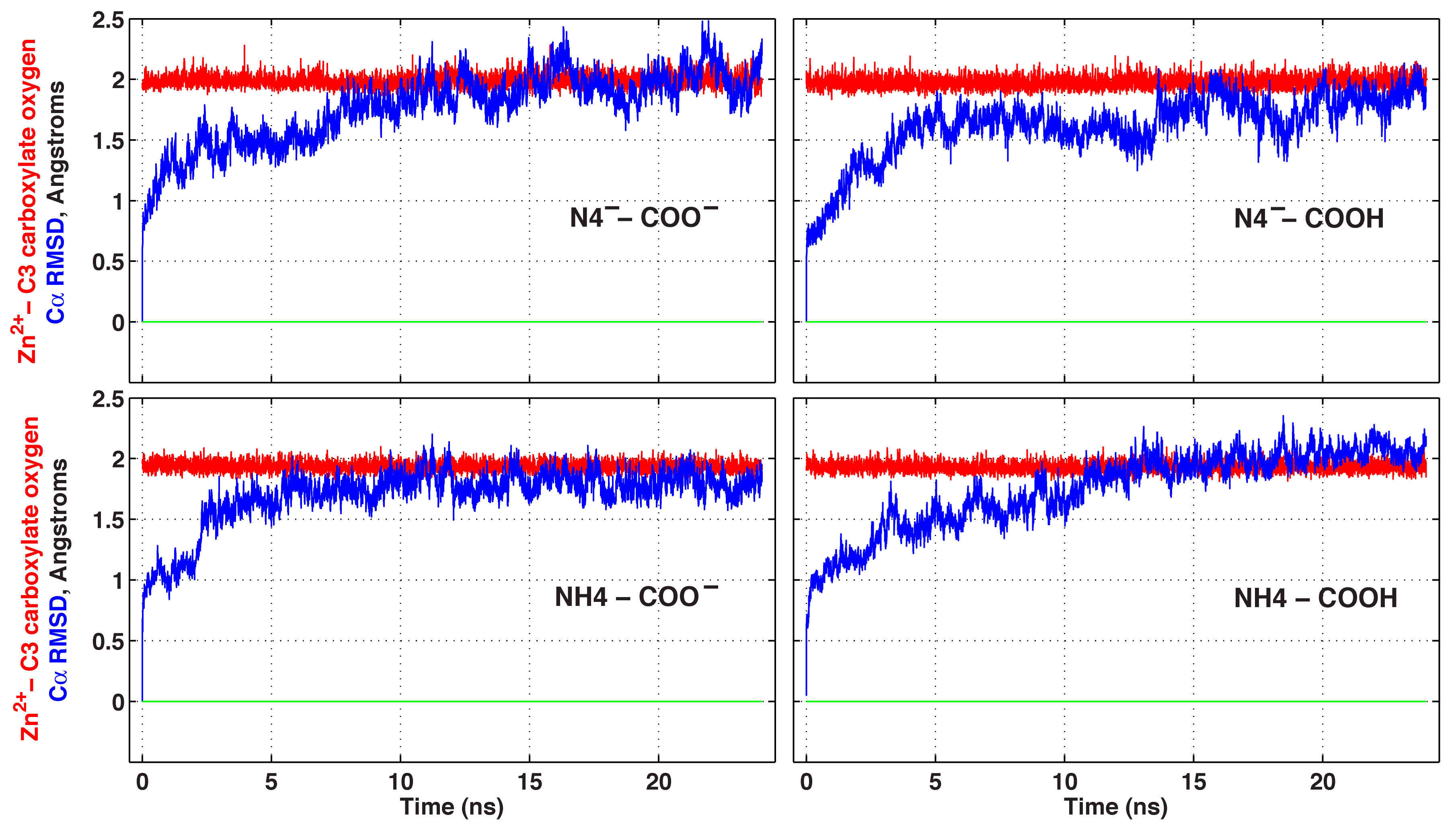

Supplement: Figure S7 — Stability of CphA and of its complex with hydrolyzed biapenem during the metadynamics simulations. In each panel, the blue trace represents the Cα-RMSD of CphA at different times with respect to time 0; the red trace is the distance between the active site Zn+2 ion and one of the oxygen atoms of the C3 carboxylate moiety of hydrolyzed biapenem. Panels A through D represent respectively the four conditions tested in metadynamics corresponding to 1) deprotonated N4 and C6 carboxylate (FES I of Fig. 9), 2) deprotonated N4 and protonated C6 carboxylate (FES II of Fig. 9), 3) protonated N4 and deprotonated C6 carboxylate (FES III of Fig. 9), 4) protonated N4 and C6 carboxylate (FES IV of Fig. 9). (TIF) [file pone.0030079.s007.tif]
