# Supplementary material for: Biapenem Inactivation by B2 Metallo β-Lactamases: Energy Landscape of the Post-Hydrolysis Reactions
Source: PLoS One. 2012 Jan 12;7(1):e30079. doi: 10.1371/journal.pone.0030079 (PMC3260057; doi:10.1371/journal.pone.0030079)
Supplement: Table S1 — Energetics of the rotation of the hydroxyethyl group of hydrolyzed biapenem. Metadynamics of biapenem in solution and in the enzyme (Configuration No. 4 in Table 1 and Fig. 6A ) were carried out starting from conformation A ( Fig. 2A ) or B ( Fig. 2B ). ΔG values are in kcal/mol. (DOC) [file pone.0030079.s008.doc]

**Table S1. Energetics of the rotation of the hydroxyethyl group of hydrolyzed biapenem.** Metadynamics of biapenem in solution and in the enzyme (Configuration No. 4 in Table 1 and Fig. 6A) were carried out starting from conformation A (**Fig. 2A**) or B (**Fig. 2B**). ΔG values are in kcal/mol.

| **Starting from conformation A** |  | ΔG^‡^(A→B) | ΔG^‡^(B→A) | ΔG^0^(A→B) |
| --- | --- | --- | --- | --- |
| Free in solution | N4^-^-COO^-^ | 2.6 | 7.6 | -5.0 |
|  | N4^-^-COOH | 12.7 | 10.7 | 2.0 |
|  | NH4-COO^-^ | 15.7 | 7.5 | 8.2 |
|  | NH4-COOH | 16.9 | 11.4 | 5.5 |
| Bound to the enzyme | N4^-^-COO^-^ | 11.4 | 13.5 | -2.0 |
|  | N4^-^-COOH | 10.9 | 17.8 | -6.9 |
|  | NH4-COO^-^ | 17.5 | 6.8 | 10.7 |
|  | NH4-COOH | 14.5 | 9.1 | 5.4 |
| **Starting from conformation B** |  | ΔG^‡^(A→B) | ΔG^‡^(B→A) | ΔG^0^(A→B) |
| Free in solution | N4^-^-COO^-^ | 4.3 | 8.8 | -4.5 |
|  | N4^-^-COOH | 10.5 | 9.0 | 1.5 |
|  | NH4-COO^-^ | 14.5 | 7.9 | 6.6 |
|  | NH4-COOH | 13.7 | 9.1 | 4.5 |
| Bound to the enzyme | N4^-^-COO^-^ | 12.5 | 15.1 | -2.5 |
|  | N4^-^-COOH | 14.0 | 17.6 | -3.5 |
|  | NH4-COO^-^ | 14.1 | 5.0 | 9.1 |
|  | NH4-COOH | 16.5 | 7.4 | 9.1 |
