# Supplementary material for: Biapenem Inactivation by B2 Metallo β-Lactamases: Energy Landscape of the Post-Hydrolysis Reactions
Source: PLoS One. 2012 Jan 12;7(1):e30079. doi: 10.1371/journal.pone.0030079 (PMC3260057; doi:10.1371/journal.pone.0030079)
Supplement: Table S2 — Sensitivity of the calculated energies to the functional used. Some of the points of the energy profiles for the cyclization reaction of hydrolyzed biapenem in solution ( Fig. 4A ) and in the enzyme (Configuration No. 4 in Table 1 and Fig. 6A ) were calculated also using the M06 functional. Energies are in kcal/mol. (DOC) [file pone.0030079.s009.doc]

Table S2. Sensitivity of the calculated energies to the functional used. Some of the points of the energy profiles for the cyclization reaction of hydrolyzed biapenem in solution (Fig. 4A) and in the enzyme (Configuration No. 4 in Table 1 and Fig. 6A) were calculated also using the M06 functional. Energies are in kcal/mol.

|  |  | ΔH^‡^ | ΔH^0^ | ΔG^‡^ | ΔG^0^ |
| --- | --- | --- | --- | --- | --- |
| NH4 solution |  | TS2/3 – RS NH4 | PS NH4 – RS NH4 | TS2/3 – RSNH4 | PS NH4 – RS NH4 |
|  | B3LYP | 19.72 | -11.94 | 21.05 | -10.41 |
|  | M06 | 22.46 | -15.14 | 22.78 | -13.34 |
| N4 solution |  | TS3 – RS NH4 | PS NH4 – RS NH4 | TS3 – RSNH4 | PS NH4 – RS NH4 |
|  | B3LYP | 13.00 | -14.46 | 13.96 | -12.36 |
|  | M06 | 12.75 | -13.35 | 12.03 | -12.22 |
| N4 enzyme 4 |  | TS3 – RS NH4 | PS NH4 – RS NH4 | TS3 – RSNH4 | PS NH4 – RS NH4 |
|  | B3LYP | 28.69 | -16.15 | 31.03 | -14.32 |
|  | M06 | 31.06 | -15.98 | 33.69 | -13.84 |
